# A network-based method for mechanistic investigation of Shexiang Baoxin Pill’s treatment of cardiovascular diseases

**DOI:** 10.1038/srep43632

**Published:** 2017-03-08

**Authors:** Hai-Yang Fang, Hua-Wu Zeng, Li-Mei Lin, Xing Chen, Xiao-Na Shen, Peng Fu, Chao Lv, Qun Liu, Run-Hui Liu, Wei-Dong Zhang, Jing Zhao

**Affiliations:** 1 Department of Mathematics, Logistical Engineering University, Chongqing, China; 2 Department of Natural Medicinal Chemistry, Second Military Medical University, Shanghai, China

## Abstract

*Shexiang Baoxin Pill* (SBP), a traditional Chinese medicine formula, is commonly used to treat cardiovascular disease (CVD) in China. However, the complexity of composition and targets has deterred our understanding of its mechanism of action. Using network pharmacology-based approaches, we established the mechanism of action for SBP to treat CVD by analyzing protein-protein interactions and pathways. The computational results were confirmed at the gene expression level in microarray-based studies. Two of the SBP’s targets were further confirmed at the protein level by Western blot. In addition, we validated the theory that SBP’s plasma absorbed compounds play major therapeutic role in treating CVD.

Cardiovascular disease (CVD) is a group of disorders that affect heart and blood vessels. It mainly includes coronary heart disease^1^, atherosclerosis^2^, myocardial ischemia^3^, myocardial fibrosis^4^, cerebrovascular disease^5^, peripheral arterial disease^6^, congenital heart disease, and deep vein thrombosis^7^,^8^. In comparison to common acute diseases (e.g., influenza), complex chronic diseases such as CVD cannot be completely cured. Drugs mainly regulate disease progression and relieve the symptoms. The treatment goal for these complex chronic diseases is to regulate the pathological state, thus controlling disease manifestation and retarding progression^9^.

*Shexiang Baoxin Pill* (SBP) is a treasured traditional Chinese medicine (TCM) formula for cardiovascular diseases. In China, SBP has a long history of clinical application to treat coronary heart disease^1^, atherosclerosis^2^, myocardial ischemia^3^ and myocardial fibrosis^4^, with significant curative effects. Earlier studies investigated SBP’s activities in cells and animal models^10^,^11^,^12^,^13^. Recent studies revealed that SBP exerts a range of pharmacological activities, including increase the level of NO^14^, enhance the expression of endothelial NO synthase^11^, alleviate damages to blood vessels^15^, decrease MMP-2 expression^16^, and promote myocardial ischemia caused by myocardial necrosis^12^. Although SBP has been extensively evaluated for clinical efficacy, its mechanism of function remains unresolved.

SBP is composed of seven medicinal materials, including Moschus, total gingenoside ginseng root, Styrax, Cinnamomi Cortex, Bufonis Venenum, Bovis Calculus Artifactus and Borneolum Syntheticum. Each ingredient contains a large number of chemical compounds. Like all TCM formulae, SBP is a multi-component and multi-target agent from the molecular perspective. As a result, its mode of action is most likely to be illustrated by network pharmacology-based approaches.

Based on the TCM formula’s characteristics of complex components, unclear targets, and holistic regulation, we have proposed a workflow for network-based TCM pharmacology study^9^. It starts with the identification of compounds present in the TCM formula and corresponding targets, and ends with discovering the signaling pathways and sub-networks regulated by the TCM formula and evaluating its effects on disease network. Several studies have been conducted following this flow diagram and revealed the modes of action for different TCM formulae^17^.

It is clear that the identification of compounds contained in TCM formula constitutes the basis of network-based TCM pharmacology study. Two different strategies have been used regarding compound selection. Some studies included all of the identified compounds in every single component of the TCM formula. For example, Zhang *et al*. studied all 451 compounds from the five Chinese herbs of the TCM formula Wu-tou decoction^18^. On the other hand, considering that most TCM formulae are taken orally, it is assumed that only the compounds that appear in blood are biologically active. Therefore, other studies only included compounds detected in the plasma after the administration of the TCM formula^19^,^20^. We used this strategy to analyze the anti-rheumatic effects of the TCM formula Huang-Lian-Jie-Du-Tang (HLJDT), in which only 14 active compounds from HLJDT were examined^21^. However, very few studies have compared the effect of plasma-absorbed compounds of a TCM formula with that of a full compound set.

This study is to investigate the mechanism of action for SBP to treat CVD using network pharmacology-based methods, and to elucidate if the plasma absorbed compounds are responsible for SBP’s anti-CVD therapeutic effects. Following extensive data mining, we assembled a list of CVD-related disease genes, two sets of SBP compounds, and their corresponding targets. The algorithm of random walk with restart (RWR) was applied to calculate the regulation scores of disease genes and target proteins to human protein-protein interaction (PPI) network and pathways, respectively. The computational results were verified by microarray-based experiments and two SBP targets were validated by Western blot.

## Materials and Methods

### Data preparation

#### CVD associated genes

DisGeNet is a plugin of Cytoscope^22^, which has collected various classes of disease associated genes. On the settings window of “Diseasegene Network” in Cytoscope, we set “select source” as “All”, “select association type” as “Any”, and “select disease class” as “Cardiovascular Disease”. In this way, we obtained 301 CVD associated genes (see Table S1 in Supplementary Material).

#### All compounds included in SBP and their corresponding targets

SBP contains seven medicinal materials that include Moschus, total ginsenoside ginseng root, Styrax, Cinnamomi Cortex, Bufonis Venenum, Bovis Calculus Artifactus and Borneolum Syntheticum. We used several TCM databases, including TCMDS^23^, HIT^24^, TCM Database@Taiwan^25^, TCMID^26^ and 3D-MSDT^27^, to collect information about all compounds in SBP. Using the name of each SBP individual medicinal material as input, we searched these databases and acquired the names and chemical structures of compounds from the corresponding medicinal ingredient as output. The outputs from these databases were combined to assemble a list of all identified SBP compounds. There are 166 chemically distinct compounds in total (see Table S2 in Supplementary Material). These are considered as full compounds included in SBP. For each compound, we searched its putative targets in the following two databases:

1. **Herbal Ingredients’ Targets Database (HIT)**^24^: HIT is a comprehensive and fully curated database that contains information on the protein targets of compounds found in Chinese herbs. Based on over 3250 papers in the literature, HIT includes 1301 protein targets affected by 586 herbal compounds found in more than 1300 Chinese herbs (http://lifecenter sgst.cn/hit/). By searching the database with the name of the chemical compound, we will be able to obtain information on its target proteins if this compound is included in the HIT.
2. **Search Tool for Interactions of Chemicals (STITCH)**^28^: STITCH is a collective database from 1133 organisms (http://stitch.embl.de/), composed of interactions existing between 300,000 small molecules and 2.6 million proteins. The biggest asset of STITCH is the huge amount of data and the structural similarity comparison tool it provides. In the event that we cannot find the targets of a compound in HIT and STITCH, we can input its structure into STITCH and identify similar chemical molecules with similarity score greater than 0.9. Targets of these chemically similar compounds are considered putative targets of the queried compound.

At the end, 522 distinct protein targets of SBP were obtained after integrating HIT and STITCH search results, in which 86 were identified from HIT, 416 were got from STITCH, and 20 are included in both of the databases (see Table S3 in Supplementary Material).

#### SBP’s plasma absorbed compounds and their targets

Our earlier serum medicinal chemistry and pharmacokinetic studies identified 34 compounds in the plasma after oral administration of SBP^14^,^29^, 26 of which were the original chemical compounds found in SBP, and 8 of which were metabolites. Among the twenty six compounds from SBP, one is from Moschus, seven are from total gingenoside ginsenoside ginseng root, eight are from Bufonis Venenum, five are from Bovis Calculus Artifactus, three are from Styrax, and two are from Borneolum Syntheticum. Mining HIT and STITCH databases, we identified 113 distinct targets for these 26 original chemical compounds. Targets of the 8 metabolites cannot be found, thus they are not pursued in this study. The detailed information is provided in Table S4 of Supplementary Material.

For convenience, we named all compounds included in SBP and SBP’s plasma absorbed compounds as SBPac and SBPpc, respectively. When studying TCM pharmacology at the molecular level, all compounds identified in a TCM formula are considered to be equivalent to the formula itself. Thus in this study, we think SBPac is equivalent to SBP itself.

#### Protein-Protein Interaction Data

Protein-protein interactions in human genome were extracted from version 9.05 of STRING^30^, a weighted interaction database with physical and functional interactions that are integrated from multiple data sources (such as experimental data, computation-based prediction, and literature mining). STRING gives each interaction a confidence score (which were normalized to the interval of [0, 1]) by a scoring system to weigh the reliability of the interaction.

In order to construct a background PPI network with high confidence edges, we first filtered the STRING with threshold 0.9. Only interactions with weight above the threshold were selected for the newly constructed background network. We also checked whether CVD disease genes and SBP’s target proteins are covered in the new network. In case of existing disease or target genes that are included in the STRING but not in the new network, we lowered the threshold for these genes and their interactions, until all these genes were included in the background network. As a result, a weighted PPI background network with 9289 nodes and 57179 edges was obtained.

#### Data of pathway gene sets

A total of 4722 pathway gene sets were derived from the C2:CP collection of MSigDB database^31^, which is a comprehensive integration of certain online pathway databases such as BioCarta, Reactome, and KEGG.

### Microarray experiment and significantly expressed genes

#### Cell lines and treatment

The cell line we used in this study is MCF7, one of the five cell lines used in connectivity map (CMAP) database as reference cells to connect biology, chemistry, and different clinical conditions including CVD^32^,^33^,^34^. MCF7 cells were obtained from American Type Culture Collection (ATCC) and maintained in MEM/EBSS (Hyclone), supplemented with 10% fetal bovine serum, 1 mM sodium pyruvate, 0.1 mM MEM non-essential amino acids, 100 unit/mL penicillin and 100 mg/mL streptomycin in a humidified environment under 5% CO_2_:95% Air at 37 °C. MCF7 cells were treated with SBP extract at 1μg/mL for 12 hours. Stock solutions of SBP were prepared in DMSO. Solvent (DMSO) treated cells were used as control, same as most microarray experiments^32^,^35^.

#### RNA isolation

Total RNA samples were extracted from MCF7 cells using Trizol reagent (Life technologies, Carlsbad, CA, US). The integrity and purity of total RNA samples were monitored by formaldehyde agarose gel electrophoresis, and quantified by spectrophotometry (NanoDrop, Wilmington, DE, USA).

#### Microarray analysis

RNA samples were purified from cells using the QIAGEN RNeasy Kit (GmBH, Germany). Biological replica was included for each cell line. cRNA products, generated from the fragmentation of double-stranded cDNA and biotin-labeled cRNA, were pooled to perform microarray experiments using Affymetrix chip (Human U133 A 2.0), by Shanghai Biotechnology Corporation following the Affymetrix technical manual.

The SBP-related microarray experiments yielded 621 differentially expressed genes, with mean expression ratios between SBPac group and control group greater than 4 or lower than 0.25. The detailed results are shown in Table S5 of Supplementary Material.

### RWR-based evaluation of drug’s effect

**Figure 1:**
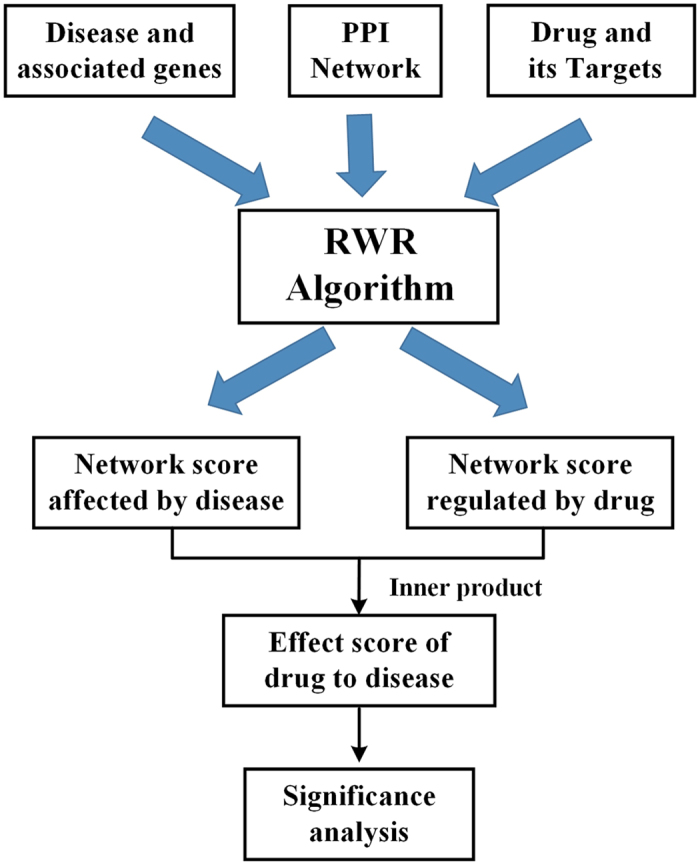
Flow diagram to evaluate the effect of a drug based on RWR.

The hypothesis for analyzing polygenic diseases and corresponding intervention in the molecule network is that genes and their products do not fulfill their biological functions independently but through prevalent interactions among different genes, and these interactions constitute a molecular network. Single gene alteration(s) incurred by diseases or drugs will affect its neighboring genes, forming a sub-network impacted by diseases or drugs. Figure 1 shows the flow diagram of determining a drug’s effect based on random walk with restart (RWR).

#### RWR algorithm

Random walk with restart (RWR) is an improved algorithm derived from random walk algorithm^36^. It simulates a random walker that starts from nodes in the seed set. At any time, the walker can either randomly move to a neighboring node from current position or return to a seed node at a relatively low probability.

RWR effectively measures the proximity of a node to seed set so that nodes adjacent to seed set can be prioritized. In view of this utility, RWR has been applied to predict potential disease genes by employing part of known disease genes to detect novel disease genes^37^,^38^,^39^. To be more specific, causative disease genes are considered as seed set while candidate genes in the PPI network are ranked based on their proximity to seed set scored by RWR in descending order. The genes with highest score are most likely to be disease-relevant.

In this study, disease genes and drug target genes were selected as seed nodes, respectively. The extent of each gene in the network affected by the disease or drug is determined in way of scoring each gene by RWR. In general, the higher score a gene receives, the more pronounced it is affected by the disease or drug. Specifically, a node’s score at the *t* + 1 step is calculated as following:


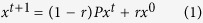


P is the column-normalized adjacent matrix of the PPI network that represents the connectivity between nodes, *x*^0^ is the initial probability vector indicating a preference to seed set, and *r* is the restart probability that generally has a low value. For a non-bipartite and undirected graph, the equation is deemed to converge. Thus, node scores can be obtained after performing the iteration in equation (1). It is obvious that the closer the nodes connected to the seed set, the higher the scores, and vice versa. The *r* in the equation (1) is a parameter that varies with practical prioritization needs. In order to choose the optimal *r*, Erten *et al*. altered *r* values to determine how RWR affects candidates’ rank^39^. It was found that an *r* value of 0.3 outperforms others. Thus, *r* denoted by 0.3 is generally adopted in the ranking of sound disease genes. We set *r* as 0.3 in this research.

#### Scoring disease’s effect on human PPI network

Scoring a disease’s effect on human PPI network is a process that estimates the proximity between each gene and disease genes by RWR algorithm. Disease-associated genes are viewed as the seed set. If there are *m* diseases genes in total, the corresponding initial component of a disease gene in equation (1) is *x*_0_(*i*) = 1 /*m*, otherwise, *x*_0_(*i*) = 0. By iterating equation (1), the steady solution *x*_d*isease*_ that represents the disease’s effect on human PPI network is obtained.

#### Scoring drug’s effect on human PPI Network

To obtain the scores measuring the effect(s) of a drug on the background network, drug target proteins are used as seed set.

If gene *i* is the target gene of active ingredients in the TCM formula, its corresponding component in the initial vector is set as *x*_0_(*i*) = 0.01, otherwise *x*_0_(*i*) = 0.

The reason for the initial vector selection is that active ingredients in TCM formula are natural products with generally weaker effects on target proteins, in comparison to chemically defined drug molecules. Moreover, our previous study found that, the FDA-approved drug has around two orders of inhibition effect on target proteins ACE and CN than the natural compound Astragaloside, when applied at the same dose^40^.

For each drug, their respective effect scores on PPI network *x*_*drug*_ are acquired with the equation (1).

In order to evaluate TCM formulae in a statistically significant fashion, we set up 1000 random counterpart sets of target proteins, each of which includes the same number of proteins randomly selected from the network. We rerun RWR process on each of these random seed sets.

#### Scoring effects of a drug to disease

In the network, the easier a node can be influenced by the seed set, the higher the score it will gain and vice versa. Therefore, genes with high scores and ranked above a defined threshold form a disease-affected sub-network that is vulnerable to diseases. Similar definition is used to establish a drug-affected sub-network. The extent of overlap between the two sub-networks is used to predict whether the drug can effectively exert its influence on the disease. This overlap can be quantitatively measured by an inner product of *x*_*disease*_ and *x*_*drug*_ so as to evaluate the performance of the drug in the treatment of the disease^41^. The equation is


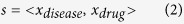


#### Scoring effects of a drug or disease on pathways

Signaling pathways are relatively independent biomolecule subsystems or interaction sub-networks that carry out important biological tasks and complex information processing between molecules. Scoring signaling pathways allows us to identify biological processes influenced by diseases or regulated by drugs.

**Figure 2:**
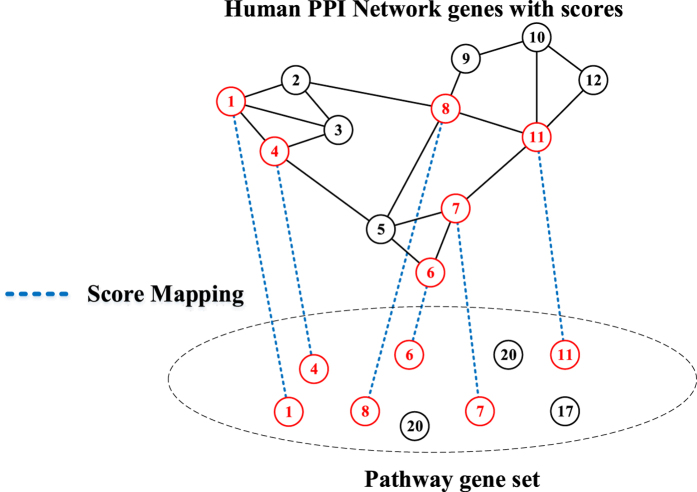
Map network genes’ scores to the pathway gene set.

As shown in Fig. 2, we score the effects of a drug or disease on pathways by mapping network scores of genes calculated onto the pathway gene sets. The details are provided below.

1. Using RWR to score the effect(s) of a disease or drug on all genes in the PPI network;
2. Mapping gene scores generated in step (1) to the corresponding genes in pathways. For genes on the pathways that are not included in the background network, their scores are assigned as zero (Fig. 2).
3. Adding up the scores in each pathway and denoting the average scores as disease’s or drug’s effect scores on the pathway.

#### Evaluation measures

To determine if the TCM formula exerts significant effect(s) on the network, we generate a wide spectrum of random seed sets (e.g., 1000 sets). Each seed set is randomly generated, containing the same number of target proteins as that of the original seed set. For each random counterpart, we compute its effect scores on each gene of the background network by equation (1).

Here Z-score is introduced to quantify the score difference existing between the original seed set and its counterparts. It is expressed as follows:


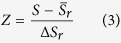


where *S* is the anti-disease effect score of the TCM formula, while 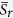 and ∆*S*_*r*_ represent mean and standard deviation of the scores stemming from random counterparts, respectively.

Then, for each disease and drug, we take pathways with highest 5% scores, respectively. These are considered as pathways significantly influenced by the disease or regulated by the drug. The common pathways influenced by the disease and drug are used to measure drug efficacy from the biological perspective.

### Target validation by Western blot

#### Drug preparation

SBP samples were ground into fine powder. Four grams of accurately weighed sample powder were transferred to a 250 mL round bottom flask, extracted with 52 mL mixed solvent (methanol: dichlormethane: water = 4: 2: 0.5, v/v) by ultrasonic extraction (30 min), filtered, and the filtrate collected. Detailed extraction method was described in the ref. 42. The filtrated extract sample was dried by rotation evaporation under 55 °C. A stock solution of 50 mg/mL was prepared by dissolving 5 mg extract sample in 100 μL DMSO.

A total number of 26 SBP compounds detected in plasma were used to prepare SBPpc. Specifically, the stock solution of SBP’s plasma absorbed compounds was prepared by dissolving the mixture of all 26 compounds in DMSO at a concentration of 100 mg/mL (see Table S6 of Supplementary Material for contents of the compounds in the solution).

#### Western blot analysis in Human Umbilical Vein Endothelial Cells

Human Umbilical Vein Endothelial Cells (HUVEC) were purchased from AllCells (Emeryville, CA, USA). The antibodies used for Western blot analysis are: rabbit anti-human intercellular adhesion molecule-1 (ICAM1, Abcam, Cambridge, MA, USA), rabbit anti-human cyclooxygenase 2 (COX-2, Cell Signaling Technology, Boston, MA, USA), and mouse anti-human GAPDH (Beyotime, Shanghai, China). Human tumor necrosis factor alpha (TNFα) was from PeproTech (Rocky Hill, NJ, USA).

HUVECs were seeded into 60 mm cell culture plates with complete endothelial growth medium (AllCells). After reaching 80% confluence, cells were exposed to SBP extract and SBPpc in the presence or absence of TNFα (25 ng/ml) for 16 hours. The conditioned media were removed, HUVECs washed and lysed, and protein concentrations determined using the BCA protein assay kit (Beyotime, Shanghai, China). The lysate samples were stored in −80 °C.

Samples containing equal amounts of proteins were separated by SDS-PAGE electrophoresis (10% SDS-polyacrylamide gel), and transferred onto polyvinyl membranes. The membranes were blocked with 5% dry milk in TRIS-buffered saline containing 0.1% Tween 20 (room temperature, 1 h), incubated with the specified primary antibodies (dilutions: COX-2 1:500, ICAM-1 1:1000, and GAPDH 1:1000) overnight at 4 °C. The membranes were visualized using Odessey (LI-COR, Lincoln, NE, USA).

## Results and Discussion

### Overlap of CVD disease genes with SBP’s target genes

From the pharmaceutical perspective, proteins encoded by disease genes may serve as potential drug targets for treatment. Earlier network analysis of the DrugBank database revealed that certain FDA approved drugs, including those that treat cardiovascular diseases, preferentially target disease genes^43^. To determine if SBP acts in a similar manner, we counted the number of CVD disease genes that are also SBP target genes. Among the 522 target genes of SBPac, 46 are CVD disease genes, which are 15.3% of the total disease genes. However, SBPpc targeted only 11 CVD disease genes, ~3.7% of the total disease genes. Table 1 summarizes the 11 common genes and their functions.

All 11 genes are associated with the functions of heart or blood vessels that are important in the etiology of CVD. Given the fact that there are 301 CVD associated genes, targeting 46 or 11 out of 301 genes is not sufficient to explain SBP’s efficacy in treating CVD.

### Network analysis of SBP on CVD

To quantitatively display the effect of SBP on CVD treatment from a network perspective, we applied RWR algorithm to measure the impact of seed nodes on other nodes in the background network.

By setting CVD disease genes, target genes of SBPac and SBPpc as seed nodes, respectively, we first used equation (1) to calculate the effect score vectors of CVD, SBPac, and SBPpc on the background network. Equation (2) was subsequently applied to calculate the inner products between affected vectors of CVD and SBPac, CVD and SBPpc. The network effect scores of SBPac and SBPpc on CVD were 0.4452 and 0.1138, respectively.

To determine whether the network effect scores of the drug to disease are significant, we compared their scores with the scores obtained from random seed sets. Based on the 522 targets of SBPac and 113 targets of SBPpc, we generated 1000 random target gene sets from the background network, respectively. Each of these sets contained the same number of targets as SBPac and SBPpc, respectively. The scores from random seed sets could be obtained using equation (1) and (2). The average and standard deviation values of effect scores for the 1000 random counterparts of each group were calculated. The Z-scores of effect scores of SBPac and SBPpc to CVD were obtained using the equation (3). As shown in Table 2, the Z-score values are 12.193 and 7.749 of SBPac and SBPpc, suggesting that the sub-networks regulated by them overlap significantly with CVD impacted sub-network. This is because a Z-score value greater than 3 often indicates a statistically significant deviation between the actual value and the random ones. Therefore, we can conclude that both SBPac and SBPpc have significant effects to CVD.

### Pathways significantly regulated by SBP

We mapped the network scores of genes from the calculation above to the 4722 pathways from the C2: CP collection of MSigDB database. For those genes on the pathways that cannot be mapped, their scores were assigned zero. The average value of all genes on each pathway was obtained. Using this approach, we acquired effect scores of CVD disease genes, SBPac and SBPpc’ target genes on each pathway, respectively.

**Figure 3:**
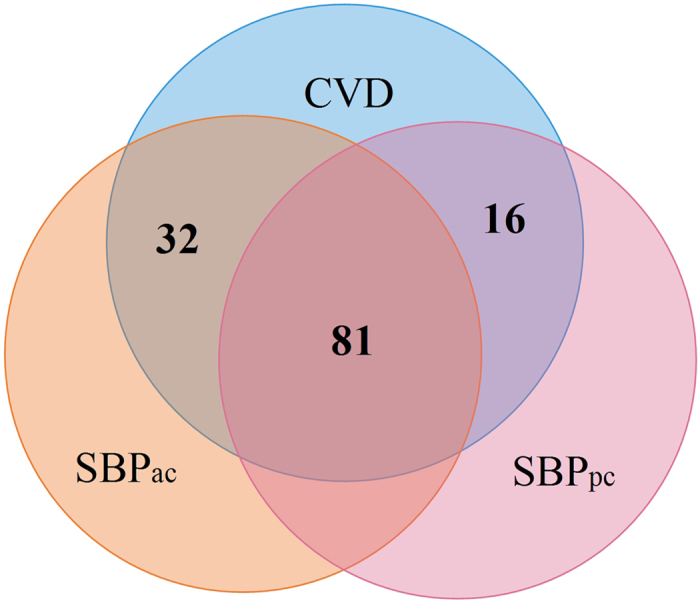
The Venn diagram of coincide pathway number from CVD, SBPac and SBPpc regulated pathways.

We defined pathways significantly regulated by the disease or drug as those with scores ranked top 5% of the 4722 pathways. This classification led to 236 pathways affected by CVD, SBPac and SBPpc, respectively. Among the 236 pathways significantly impacted by CVD, 113 and 97 are also regulated by SBPac and SBPpc (Fig. 3), constituting 47.8% and 41% of the CVD pathways, respectively. The observation that nearly half of CVD affected pathways are regulated by SBP supports the therapeutic effect of SBP on CVD.

There are 81 pathways that are significantly affected by CVD and regulated by SBPac and SBPpc both, constituting 71.7% of the SBPac regulated CVD affected pathways. Since SBPac is considered as equivalent to SBP itself, these results suggest that, SBP’s plasma absorbed compounds play major roles in SBP’s treatment of CVD.

### Microarray experiment validation

Microarray-based studies were performed to verify the network analysis results. A total of 621 genes with mean expression ratios of SBP group to control group greater than 4 or less than 0.25, were considered differentially expressed genes.

Among these 621 genes, only 31 and 9 genes are target genes of SBPac and SBPpc, respectively, identified by the bioinformatics method. Meanwhile, 29 of the 621 genes are CVD disease genes, among which, two genes ICAM1 and COX-2 are also target genes of SBPac and SBPpc. Both ICAM1 and COX-2 are important disease genes associated with CVD.

The 621 significantly expressed genes were set as the seed nodes and the corresponding component of the gene in the initial vector as normalized differential expression ratio. Using approach similar to that described earlier, we calculated the differentially expressed genes’ network effect scores on CVD and on each of the 4722 pathways from the C2: CP collection of MSigDB database. This led to the identification of 236 pathways significantly regulated by these 621 genes with scores in the top 5%. Among them, 119 pathways are affected by CVD, which is 50.4% of all the 236 pathways. These gene expression data further support the therapeutic effect of SBP on CVD.

We compared the 113 and 97 CVD affected pathways, which are also regulated by SBPac and SBPpc, with the 119 CVD affected pathways regulated by significantly expressed genes under SBP treatment, respectively. It was found that 80 of the 113 SBPac affected pathways and 70 of the 97 SBPpc affected pathways also appeared in these 119 pathways, corresponding to 67.2% and 58.8%, respectively. Specifically, 63 CVD affected pathways are also regulated by SBPac, SBPpc, and altered gene expression significantly upon SBP treatment. These results validate the reliability of SBP’s target genes and their regulating pathways identified by our bioinformatics methods.

**Figure 4:**
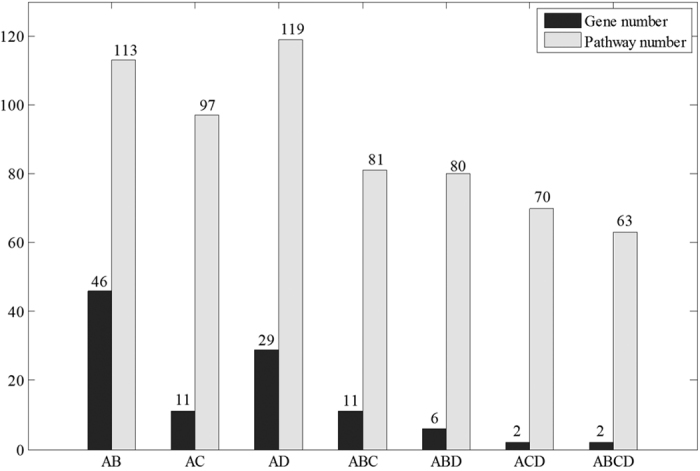
Comparison of the gene number in different intersection sets of the four gene sets and the number of pathways affected by corresponding genes, where (A, B, C and D) denote CVD disease genes, SBPac’s target genes, SBPpc’s target genes, and significantly expressed genes upon SBP treatment, respectively. AB denotes the intersection set between A and B, and so on.

Figure 4 shows the number of genes in different intersection sets of the four gene sets, and the number of pathways affected by genes in these intersections. It could be seen that, although SBP directly act on only a few CVD disease genes, through network interactions, SBPac, SBPpc, and significantly expressed genes under SBP treatment regulate a greater number of common CVD-related signaling pathways. Although the 11 CVD genes targeted by SBPpc (AC in Fig. 4) are only 24% of the 46 CVD genes targeted by SBPac (AB in Fig. 4), the number of CVD associated pathways regulated by SBPpc (AC in Fig. 4, 97) is 86% of the number of the CVD associated pathways regulated by SBPac (AB in Fig. 4,113). Meanwhile, only few CVD disease genes targeted by SBPac and SBPpc were validated in the gene expression experiment, i.e., 6 and 2 genes, respectively (ABD and ACD in Fig. 4). In contrast, more CVD affecting pathways regulated by SBPac and SBPpc were verified in the gene expression study, i.e., 80 and 70 pathways, respectively (ABD and ACD in Fig. 4). That is to say, about 70% CVD associated pathways affected by SBPac and SBPpc were validated in the microarray-based studies.

These results suggest that SBP regulates CVD associated network through multi-components and multi-targets to achieve its clinical efficacy. The high consistency between the pathways identified by the bioinformatics methods and the microarray confirmation studies exemplifies the reliability of our data mining and analytical methods. It also reveals that SBP’s plasma absorbed components exert nearly the same function as that of SBP’s full compound set for CVD treatment.

Among the 63 experimentally validated CVD pathways regulated by both SBPac and SBPpc (ABCD in Fig. 4, Table S7 of Supplementary Material), most are directly associated with CVD, further supporting the effectiveness of SBP to treat CVD. Table 3 lists some of these pathways that are associated with CVD.

### Target validation in cell-based studies

Based on above analysis, genes ICAM-1 and COX-2 are included in four different gene sets under study, *i.e*., CVD disease genes, SBPac’s target genes, SBPpc’s target genes, and significantly expressed genes under SBP treatment. Moreover, their encoded proteins are readily available. Thus we chose protein ICAM-1 and COX-2 for experimental validation.

**Figure 5:**
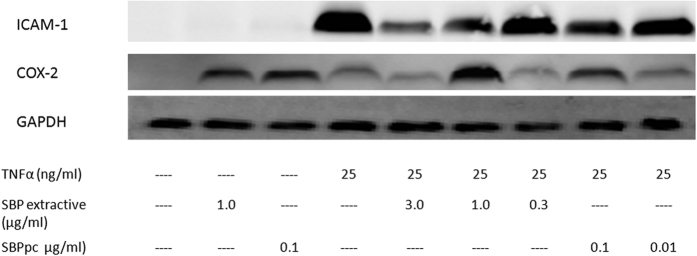
Expression of ICAM-1 and COX-2 proteins in HUVECs treated with SBP extract and SBPpc samples for 16 hours in the presence or absence of TNFα (25 ng/ml). Specific proteins were detected by Western blot as described in the Material and Methods. Each blot is representative of three independent experiments.

To validate SBP’s effects on ICAM-1 and COX-2, we conducted Western blot experiment in HUVECs. As shown in Fig. 5, SBP extract (1 μg/ml) and SBPpc (0.1 μg/ml) each induced the expression of COX-2 protein while inhibiting the expression of TNFα-induced ICAM-1 protein.

Both ICAM-1 and COX-2 are expressed in endothelial cells and play important roles in the initiation and progression of cardiovascular disease. ICAM-1 facilitates the transmigration of leukocytes across vascular wall during inflammatory response. COX-2 converts arachidonic acid to various metabolites, including prostacyclin (PGI2), a major COX-2 metabolite found in endothelial cells and an effective vasodilator. These observations support the notion that SBP and its plasma absorbed compounds (SBPpc) exert their anti-CVD effects by vasodilation and anti-inflammatory action, through the up-regulation of COX-2 and down-regulation of ICAM-1.

## Conclusions

This study investigated the therapeutic effect of SBP on CVD in depth using a network pharmacological approach. First, we calculated the network effect score of disease and drug on genes in the background network and further analyzed the score’s significance to determine whether SBP significantly impacts CVD. Second, we mapped the scores of all genes in the background network obtained through RWR, to 4722 pathways and calculated the regulation scores of CVD, all compounds of SBP and plasma absorbed compounds of SBP to these pathways. As a result, we identified biological processes significantly regulated by SBP’s different compound sets and found most of them are associated with CVD. Finally, we validated our bioinformatics results in microarray- and Western blot-based studies.

We found that, unlike FDA approved CVD drugs that mainly target specific disease genes, SBP only targets a very small fraction of CVD disease genes. However, from the perspective of network regulation, the sub-network regulated by SBP and most signaling pathways significantly regulated by SBP are overlapped with those influenced by CVD disease genes, respectively. These results suggest that SBP achieves its efficacy on CVD treatment by regulating the disease network though interactions between genes, instead of directly acting on CVD disease genes. When compared to the anti-CVD effects of all compounds included in SBP, SBP’s plasma absorbed compounds acquired significant anti-CVD effect score. Approximately 70% of CVD associated pathways affected by both SBP’s full compound set and SBP’s plasma absorbed compound set were validated in microarray-based studies, indicating that SBP’s plasma absorbed compounds play a major role in SBP’s treatment of CVD. Finally, two important CVD associated disease genes that are targeted by SBP were validated at the protein expression level by Western blot.

In summary, this study applied a network pharmacology approach to determine SBP’s anti-CVD effect. It shed lights on the modern approaches to investigate TCM pharmacology and to promote the development of traditional medicine.

## Additional Information

**How to cite this article**: Fang, H.-Y. *et al*. A network-based method for mechanistic investigation of Shexiang Baoxin Pill’s treatment of cardiovascular diseases. *Sci. Rep.* **7**, 43632; doi: 10.1038/srep43632 (2017).

**Publisher's note:** Springer Nature remains neutral with regard to jurisdictional claims in published maps and institutional affiliations.

## Supplementary Material

Supplementary Material

## Figures and Tables

**Table 1:** CVD disease genes targeted by SBP’s plasma absorbed compounds (SBPpc).

No	Gene Symbol	Gene function
1	COL1A1	Platelet-derived growth factor binding protein^44^
2	CYP2C9	Heme binding protein^45^
3	ESR2	A group of heme-thiolate monooxygenases^46^
4	ICAM1	Ligands for the leukocyte adhesion protein LFA-1 (integrin alpha-L/beta-2)^47^
5	LDLR	Receptor of low-density lipoprotein (plasma cholesterol apolipoprotein)^48^
6	MMP9	May play an essential role in local proteolysis of the extracellular matrix and in leukocyte migration^49^.
7	NOS3	Modulate angiogenesis, blood pressure, and calcium channel^50^
8	COX-2	Regulate vasoconstriction and blood pressure^51^
9	TGFB1	Regulation of vascular endothelial cell migration^52^
10	TLR4	Immune response and inflammatory process regulation^53^
11	VCAM1	Involved in calcium-mediated signaling pathways of intracellular calcium source^54^

**Table 2:** The effect scores of SBPac and SBPpc on CVD.

Name	Target number	Effect score	Z-score
SBP’s all compounds (SBPac)	522	0.4452	12.1930
SBP’s plasma absorbed compounds (SBPpc)	113	0.1138	7.7493

**Table 3:** List of selected CVD affecting pathways regulated by both SBP and SBP’s plasma absorbed components.

ID	Pathway Name	The association with CVD
1	AT1R_PATHWAY	Vasodilator and myocardial hypertrophy^55^
2	SPPA_PATHWAY	Vasoconstriction and platelet activation
3	EPO_PATHWAY	Increasing erythrocyte^56^
4	HIF_PATHWAY	Vascular remodeling and thrombopoietin^57^
5	IL4_PATHWAY	Hematopoietic association^58^
6	IL6_PATHWAY	Hematopoietic association^59^
7	CARDIACEGF_PATHWAY	Cardiac hypertrophy and angiotensin^60^
8	TPO_PATHWAY	Thrombopoietin^61^
9	ANGIOPOIETINRECEPTOR_PATHWAY	Angiopoietin^62^
10	S1P_S1P1_PATHWAY	Endothelial cell induction^63^
11	VEGFR1_PATHWAY	Vascular endothelial growth factor receptor^64^
12	LYMPHANGIOGENESIS_PATHWAY	Angiogenesis^65^
13	HEMATOPOIESIS_STAT3_TARGETS	Hematopoietic^66^
