# Supplementary material for: A network-based method for mechanistic investigation of Shexiang Baoxin Pill’s treatment of cardiovascular diseases
Source: Sci Rep. 2017 Mar 8;7:43632. doi: 10.1038/srep43632 (PMC5341564; doi:10.1038/srep43632)
Supplement: Supplementary Material [file srep43632-s1.pdf]

Supplementary Information

A network-based method for mechanistic investigation of Shexiang Baoxin Pill's treatment of cardiovascular diseases

Hai-Yang Fang[1 §], *Hua-Wu Zeng*[2 §], *Li-Mei Lin*[1], *Xing Chen*[1], *Xiao-Na Shen*[1], *Peng Fu*[2], *Chao Lv*[2], *Qun Liu*[2], *Run-Hui Liu*[*2], *Wei-Dong Zhang*[*2] *and Jing Zhao*[*1,2]

[1]Department of Mathematics, Logistical Engineering University, Chongqing, China

[2]Department of Natural Medicinal Chemistry, Second Military Medical University, Shanghai, China

[§]These authors contributed equally to this work

* **Corresponding author**: Jing Zhao: zhaojanne@gmail.com, Wei-dong Zhang: wdzhangy@hotmail.com, and Run-hui Liu: lyliurh@126.com

Contents

Table S1. Genes associated with CVD
Table S2. Chemical compounds of SBP
Table S3. SBP's target proteins
Table S4. SBP's plasma absorbed compounds and their targets
Table S5. Significantly expressed genes and corresponding fold change values
Table S6. Content of the 26 bioactive compounds in the SBPpc solution
Table S7. The 63 experimentally validated CVD pathways regulated by both SBPac and SBPpc

Table S1 Genes associated with CVD

Entrez ID	GENE Symbol
26298	EHF
338	APOB
197	AHSG
114805	GALNT13
3553	IL1B
390174	OR9G1
4837	NNMT
7980	TFPI2

441911	OR10J3
5465	PPARA
283189	OR9G4
2646	GCKR
5139	PDE3A
339479	FAM5C
9201	DCLK1
57698	KIAA1598
118	ADD1
51083	GAL
56259	CTNNBL1
6364	CCL20
151	ADRA2B
1907	EDN2
1524	CX3CR1
5743	PTGS2
4023	LPL
57761	TRIB3
2696	GIPR
5791	PTPRE
2161	F12
54873	PALMD
10911	UTS2
2688	GH1
84059	GPR98
3479	IGF1
1401	CRP
9619	ABCG1
6696	SPP1
9955	HS3ST3A1
10221	TRIB1
2876	GPX1
8858	PROZ
120406	FAM55B
3992	FADS1
3569	IL6
3784	KCNQ1
116519	APOA5
2894	GRID1
4313	MMP2
337	APOA4
7450	VWF
221692	PHACTR1

2695	GIP
11132	CAPN10
91137	SLC25A46
63826	SRR
7124	TNF
7054	TH
22926	ATF6
3484	IGFBP1
54769	DIRAS2
285195	SLC9A9
2243	FGA
2099	ESR1
348	APOE
9365	KL
153241	CEP120
10891	PPARGC1A
6462	SHBG
10257	ABCC4
2056	EPO
4288	MKI67
3557	IL1RN
81833	SPACA1
1435	CSF1
3673	ITGA2
4153	MBL2
26059	ERC2
6774	STAT3
22843	PPM1E
585	BBS4
1559	CYP2C9
167465	ZNF366
5169	ENPP3
183	AGT
4852	NPY
4056	LTC4S
128821	CST9L
5950	RBP4
5739	PTGIR
1002	CDH4
7412	VCAM1
2852	GPER
1813	DRD2
3600	IL15

9575	CLOCK
814	CAMK4
5328	PLAU
128822	CST9
129684	CNTNAP5
60412	EXOC4
4524	MTHFR
2159	F10
9415	FADS2
6144	RPL21
1113	CHGA
5480	PPIC
7048	TGFBR2
23310	NCAPD3
4016	LOXL1
4878	NPPA
3667	IRS1
79001	VKORC1
1234	CCR5
2212	FCGR2A
2897	GRIK1
255520	ELMOD2
6403	SELP
4881	NPR1
53942	CNTN5
2152	F3
5891	MOK
5144	PDE4D
57580	PREX1
2786	GNG4
23118	TAB2
2784	GNB3
1012	CDH13
152330	CNTN4
1278	COL1A2
3039	HBA1
91074	ANKRD30A
5142	PDE4B
376497	SLC27A1
1585	CYP11B2
3576	IL8
8091	HMGA2
7351	UCP2

284040	CDRT4
5627	PROS1
1952	CELSR2
222553	SLC35F1
104	ADARB1
6347	CCL2
5468	PPARG
51741	WWOX
57531	HACE1
55720	TSR1
2100	ESR2
23345	SYNE1
3630	INS
6720	SREBF1
54332	GDAP1
4208	MEF2C
6401	SELE
3123	HLA-DRB1
185	AGTR1
1557	CYP2C19
7040	TGFB1
3586	IL10
9745	ZNF536
55247	NEIL3
25924	MYRIP
213	ALB
26047	CNTNAP2
154	ADRB2
2138	EYA1
1404	HAPLN1
6331	SCN5A
127385	OR10J5
2168	FABP1
57863	CADM3
79068	FTO
1805	DPT
325	APCS
3084	NRG1
2244	FGB
23671	TMEFF2
3240	HP
9370	ADIPOQ
257194	NEGR1

7881	KCNAB1
177	AGER
56606	SLC2A9
54914	KIAA1797
7839	LSL
133	ADM
22795	NID2
8792	TNFRSF11A
3297	HSF1
3952	LEP
10060	ABCC9
948	CD36
23263	MCF2L
1471	CST3
4018	LPA
9771	RAPGEF5
55703	POLR3B
2200	FBN1
4846	NOS3
160777	CCDC60
3690	ITGB3
23293	SMG6
7143	TNR
7421	VDR
4982	TNFRSF11B
9388	LIPG
5327	PLAT
153	ADRB1
2952	GSTT1
5314	PKHD1
9962	SLC23A2
54212	SNTG1
84236	RHBDD1
345	APOC3
3753	KCNE1
7391	USF1
5350	PLN
2878	GPX3
6262	RYR2
6558	SLC12A2
10516	FBLN5
875	CBS
387119	CEP85L

58191	CXCL16
7099	TLR4
26476	OR10J1
5054	SERPINE1
219493	OR5AR1
2155	F7
7941	PLA2G7
2266	FGG
8600	TNFSF11
64241	ABCG8
9312	KCNB2
1906	EDN1
5444	PON1
3762	KCNJ5
84722	PSRC1
6532	SLC6A4
9353	SLIT2
3383	ICAM1
3077	HFE
3329	HSPD1
6288	SAA1
79991	OBFC1
2053	EPHX2
3075	CFH
6638	SNRPN
9631	NUP155
726	CAPN5
341	APOC1
5742	PTGS1
2169	FABP2
2205	FCER1A
54504	CPVL
3757	KCNH2
79698	ZMAT4
55668	C14orf118
27303	RBMS3
5445	PON2
137492	VPS37A
5992	RFX4
1573	CYP2J2
192343	NEWENTRY
7486	WRN
1071	CETP

3481	IGF2
164781	WDR69
7408	VASP
5781	PTPN11
7139	TNNT2
1047	CLGN
9948	WDR1
161357	MDGA2
7422	VEGFA
131096	KCNH8
3570	IL6R
64410	KLHL25
10217	CTDSPL
1356	CP
4314	MMP3
54433	GAR1
1636	ACE
335	APOA1
11107	PRDM5
10082	GPC6
1277	COL1A1
4318	MMP9
6843	VAMP1
3606	IL18
9722	NOS1AP
255738	PCSK9
1520	CTSS
9905	SGSM2
338675	OR5AP2
10003	NAALAD2
6894	TARBP1
3949	LDLR
6649	SOD3
56729	RETN
4879	NPPB

Table S2. SBP's medicinal materials and chemical compounds

Medicinal material	Chemical compound
Moschus	Muscone
	Normuscone
	Muscopyridine
	Muscol

	Muscopyran
	Hydroxymuscopyridine A
	Hydroxymuscopyridine B
	3-Methylcyclotridecan-1-One
	Cyclotetradecanone
	Cholest-4-En-3-One
	Cholesterol
	Testosterone
	Estradiol
	5 Alpha-Androstan-3,17-Dione
Total gingenoside ginseng root	Panaxadiol
	Panaxatrio1
	Oleanolic Acid
	Ginsenoside Fc
	Ginsenoside Ra1
	Ginsenoside Ra2
	Ginsenoside Ra3
	Ginsenoside Rb1
	Ginsenoside Rb2(20-[(6-O-Alpha-L-Arabinopyranosyl-Beta-D-Glucopyranosyl)Oxy]-12Beta-Hydroxydammar-24-En-3Beta-Yl 2-O-Beta-D-Glucopyranosyl-Beta-D-Glucopyranoside)
	Ginsenoside Rb3(Dammarane,B-D-Glucopyranoside Deriv)
	Ginsenoside Rd
	Ginsenoside Rc
	Ginsenoside Re
	Ginsenoside Rf(Dammarane,B-D-Glucopyranoside Deriv)
	Ginsenoside Rg1
	Ginsenoside Rg2
	Ginsenoside Rg3
	Ginsenoside Rh1
	Ginsenoside Rh2
	Ginsenoside Ro
	Ginsenoside Rs1
	Ginsenoside Rs2
	Notoginsenoside R1
	Notoginsenoside R2
	Notoginsenoside Fa
	Quinquenoside R1
Bufonis Venenum	Cinobufagin 3-Acetate
	Resibufogenin
	Resibufagin

	Cinobufagin
	1-Hydroxy-Cinobufagin
	Gamabufotalin(Gamabufogenin)
	Arenobufagin
	Bufalin
	1B-Hydroxybufalin
	Bufotalin
	Bufarenogin
	Ψ-Bufarenogin(Psi-Bufarenogin)
	Desacetylbufotalin
	Telocinobufagin
	Cinobufotalin
	19-Oxo-Desacetyl-Cinobufotalin
	Resibufogenol
	Cinobufaginol
	19-Oxo-Cinobufotalin
	Marinobufagenin
	Desacetyl-Cinobufagin
	Desacetylcinobufaginol
	Bufotalidin(Hellebrigenin)
	Bufotalinin
	Marinobufagin
	Nicotinamide
	Butanoic Acid
	Bufoserotonins A
	Bufoserotonins B
	Bufoserotonins C
	2-Piperidinecarboxylic Acid
	6-Oxo-Methylester
	Adenine
	Uracil
	5-Hydroxyindoleacetic Acid (5-Hiaa)(5-Hydroxyindole-3-Acetic Acid)
	Bufothionine
Bovis Calculus Artifactus	Ursodeoxycholic Acid
	Cholic Acid
	Deoxycholic Acid
	Chenodeoxycholic Acid
	Hyodeoxycholic Acid
	Taurocholic Acid
	Glycocholic Acid
	Cholesterol
	Bilirubin

Styrax	Benzyl Benzoate(Ascabin)
	Oleanonic Acid
	3-Epioleanolic Acid
	Pimaric Acid
	Isopimaric Acid
	Dehydroabietic Acid
	Abietatriene-3B-Ol
	Methyl 4-Hydroxycinnamate(Methyl P-Couma)
	Vanillin
	Vanillic Acid
	5-Hydroxymethyl-2-Furaldehyde
	Cinnamyl Acetate
	Benzyl Cinnamate
	Alpha-Pinene
	Beta-Pinene
	Myrcene
	Camphene
	Limonene
	L, 8-Cineole
	P-Cymene
	Terpinolene
	Linalool
	4-Terpineol
	A-Terpineol
	Cinnamaldehyde
	4-Ethyphenol
	2-Ethyphenol
	3-Ethyphenol
	Allylphenol
	N-Propyl Cinnamate
	B-Phenylpropionic Acid
	L-Benzoyl-3-Phenylpropyne
	Benzoic Acid
	Palmitic Acid
	Linoleic Acid
	Dihydrocoumarone
	Epoxycinnamyl Cinnamate
	Cis-Cinnamic Acid
	Cis-Cinnamylcinnamate
Cinnamomi Cortex	Cinnamaldehyde
	Cinnamyl Acetate
	Ethylcinnamate
	Benzyl Benzoate

	Benzaldehyde
	Coumarin
	B-Cadinene
	Calamenene
	B-Elemane
	Protocatechuic Acid
	Transcinnamic Acid
	3'-○-Methyl-(-)-Epicatechin
	5,3'-Di-O-Methylate-(-)-Epicatechin
	5,7,3'-Tri-O-Methylate-(-)-Epicatechin
	(4'-○-Methyl-(+)-Catechin)
	(7,4'-Di-O-Methylate-(+)-Catechin)
	(5,7,4'-Tri-O-Methylate-(+)-Catechin)
	((-)-Epicatechin 3-O-B-D-Glucopyranoside)
	((-)-Epicatechin 8-C-B-D-Glucopyranoside)
	((-)-Epicatechin 6-C-B-D-Glucopyranoside)
	(-)-Epicatechin
	Cinnamtannin A2
	Cinnamtannin A3
	Procyanidin
	(Procyanidin B-2 6-C-B-D-Glucopyranoside)
	Cinnzeylanine
	Cinncassiols
	Cinncassiols A
	Cinncassiols B
	Cinncassiols C3
	Cinncassiols D1
	Cinncassiols D3
	Cinncassiols D4
	Lyoniresinol-3A-O-B-D-Glucopyranoside
	3,4,5-Trimethoxyphenol-B-D-Apiofuranosyl(1→6)-B-D-Glucopyranoside
	Syringaresinol
	5,7-Dimethyl-3',4'-Di-○-Methylene-(±)-Epicatechin
	Cinnamic Aldehydecyclicglycerol-1,3-Acetal(9,2'-Trans)
	Cinnamic Aldehydecyclicglycerol-1,3-Acetal(9,2'-Cis)
	Cassioside
	Cinnamoside
Borneolum Syntheticum	D-Borneol
	Isoborneol

Table S3. SBP's target proteins

Compounds	Gene Symbol	Entrez	Database
β-phenylpropionic acid	GOT1	2805	STITCH
β-phenylpropionic acid	GOT2	2806	STITCH
β-phenylpropionic acid	HADHA	3030	STITCH
β-phenylpropionic acid	LPO	4025	STITCH
β-phenylpropionic acid	MPO	4353	STITCH
β-phenylpropionic acid	EPX	8288	STITCH
β-phenylpropionic acid	GOT1L1	137362	STITCH
β-elemane	LTA4H	4048	STITCH
β-cadinene	TRIO	7204	STITCH
β-cadinene	KALRN	8997	STITCH
β-cadinene	ARHGEF25	115557	STITCH
Vanillin,Vanillic acid,cis-cinnamic acid,cis-cinnamylcinnamate,3'-○-methyl -(-)-epicatechin,5,3'-di-O-methylate-(-)-epicatechin,5,7,3'-tri-O-methylate-(-)-epicatechin,〔4'-○-methyl-(+)-catechin〕,（7,4'-di-O-methylate-(+)-catechin）,（5,7,4'-tri-O-methylate-(+)-catechin）,5，7-dimethyl-3'，4'-di-○-methylene-(±)-epicatechin	CA2	760	STITCH
Vanillin,cis-cinnamic acid,cis-cinnamylcinnamate,3'-○-methyl -(-)-epicatechin,5,3'-di-O-methylate-(-)-epicatechin,5,7,3'-tri-O-methylate-(-)-epicatechin,〔4'-○-methyl-(+)-catechin〕,（7,4'-di-O-methylate-(+)-catechin）,（5,7,4'-tri-O-methylate-(+)-catechin）,5，7-dimethyl-3'，4'-di-○-methylene-(±)-epicatechin	CA1	759	STITCH
Vanillin	OR1G1	8390	STITCH
Vanillin	TRPV3	162514	STITCH
Ursodeoxycholic acid,Cholic acid,Glycocholic acid	FABP6	2172	HIT,STITCH
Ursodeoxycholic acid	E2F1	1869	HIT
Ursodeoxycholic acid	NCOA1	8648	HIT
uracil	CD69	969	STITCH
uracil	CDA	978	STITCH
uracil	DPYD	1806	STITCH
uracil	DPYS	1807	STITCH
uracil	TYMP	1890	STITCH
uracil	GPR17	2840	STITCH

uracil	TDG	6996	STITCH
uracil	TYMS	7298	STITCH
uracil	UNG	7374	STITCH
uracil	UPP1	7378	STITCH
uracil	CCNO	10309	STITCH
uracil	SMUG1	23583	STITCH
uracil	UCKL1	54963	STITCH
uracil	AICDA	57379	STITCH
uracil	PUS3	83480	STITCH
uracil	UPRT	139596	STITCH
uracil	TRUB1	142940	STITCH
uracil	UPP2	151531	STITCH
Testosterone,estradiol,Ginsenoside Fc,Ginsenoside Ra1,Ginsenoside Ra2,Ginsenoside Ra3,Ginsenoside Rb2(20-[(6-O-alpha-L-arabinopyranosyl-beta-D-glucopyranosyl)oxy]-12beta-hydroxydammar-24-en-3beta-yl 2-O-beta-D-glucopyranosyl-beta-D-glucopyranoside),Ginsenoside Rb3(Dammarane,b-D-glucopyranoside deriv),Ginsenoside Rs1,Ginsenoside Rs2,Notoginsenoside Fa,Quinquenoside R1,(-)-epicatechin,cinnamtannin A2,cinnamtannin A3,（procyanidin B-2 6-C-β-D-glucopyranoside）,cinncassiols C3,cinnamoside	AKT1	207	STITCH,HIT
Testosterone,estradiol,(-)-epicatechin	CYP19A1	1588	STITCH,HIT
Testosterone,estradiol,(-)-epicatechin	ABCG2	9429	STITCH,HIT
Testosterone,estradiol	IGF1	3479	STITCH
Testosterone,estradiol	IGFBP3	3486	STITCH
Testosterone,estradiol	PRL	5617	STITCH
Testosterone,estradiol	SHBG	6462	STITCH
Testosterone,Bufoserotonins A,Bufoserotonins B	POMC	5443	STITCH
Testosterone,bilirubin,D-borneol	UGT1A1	54658	STITCH
Testosterone,bilirubin	UGT1A6	54578	STITCH
Testosterone	HSD3B1	3283	STITCH
Testosterone	HSD17B3	3293	STITCH
Testosterone	HSD17B6	8630	STITCH
Taurocholic acid,(-)-epicatechin	JUN	3725	HIT,HIT
Taurocholic acid	GSR	2936	HIT
Taurocholic acid	NR3C2	4306	HIT

Taurocholic acid	VEGFA	7422	HIT
syringaresinol	NOS1	4842	STITCH
Sulfadiazine	BRD2	6046	STITCH
resibufogenin,Resibufagin,gamabufotalin(Gamabufogenin),1β-Hydroxybufalin,bufotalin,19-oxo-desacetyl-cinobufotalin,resibufogenol,cinobufaginol,Desacetyl-cinobufagin,Desacetylcinobufaginol,Bufotalinin	SERPINA6	866	STITCH
resibufogenin,Resibufagin,gamabufotalin(Gamabufogenin),1β-Hydroxybufalin,bufotalin,19-oxo-desacetyl-cinobufotalin,resibufogenol,cinobufaginol,Desacetyl-cinobufagin,Desacetylcinobufaginol,Bufotalinin	DRG1	4733	STITCH
resibufogenin,Resibufagin,gamabufotalin(Gamabufogenin),1β-Hydroxybufalin,bufotalin,19-oxo-desacetyl-cinobufotalin,resibufogenol,cinobufaginol,Desacetyl-cinobufagin,Desacetylcinobufaginol,Bufotalinin	PGK1	5230	STITCH
resibufogenin,Resibufagin,gamabufotalin(Gamabufogenin),1β-Hydroxybufalin,bufotalin,19-oxo-desacetyl-cinobufotalin,resibufogenol,cinobufaginol,Desacetyl-cinobufagin,Desacetylcinobufaginol,Bufotalinin	PHKA1	5255	STITCH
resibufogenin,Resibufagin,gamabufotalin(Gamabufogenin),1β-Hydroxybufalin,bufotalin,19-oxo-desacetyl-cinobufotalin,resibufogenol,cinobufaginol,Desacetyl-cinobufagin,Desacetylcinobufaginol,Bufotalinin	RPS12	6206	STITCH
resibufogenin,Resibufagin,gamabufotalin(Gamabufogenin),1β-Hydroxybufalin,bufotalin,19-oxo-desacetyl-cinobufotalin,resibufogenol,cinobufaginol,Desacetyl-cinobufagin,Desacetylcinobufaginol,Bufotalinin	TAF1	6872	STITCH
resibufogenin,Resibufagin,gamabufotalin(Gamabufogenin),1β-Hydroxybufalin,bufotalin,19-oxo-desacetyl-cinobufotalin,resibufogenol,cinobufaginol,Desacetyl-cinobufagin,Desacetylcinobufaginol,Bufo	ECEL1	9427	STITCH

talinin			
resibufogenin,Resibufagin,gamabufotalin(Gamabufogenin),1β-Hydroxybufalin,bufotalin,19-oxo-desacetyl-cinobufotalin,resibufogenol,cinobufaginol,Desacetyl-cinobufagin,Desacetylcinobufaginol,Bufotalinin	GCN1L1	10985	STITCH
resibufogenin,Resibufagin,gamabufotalin(Gamabufogenin),1β-Hydroxybufalin,bufotalin,19-oxo-desacetyl-cinobufotalin,resibufogenol,cinobufaginol,Desacetyl-cinobufagin,Desacetylcinobufaginol,Bufotalinin	RWDD1	51389	STITCH
resibufogenin,Resibufagin,gamabufotalin(Gamabufogenin),1β-Hydroxybufalin,bufotalin,19-oxo-desacetyl-cinobufotalin,resibufogenol,cinobufaginol,Desacetyl-cinobufagin,Desacetylcinobufaginol,Bufotalinin	ZC3H15	55854	STITCH
protocatechuic acid,transcinnamic acid	MGA	23269	HIT
protocatechuic acid	PRKACA	5566	HIT
protocatechuic acid	PRKCA	5578	HIT
protocatechuic acid	PRKCG	5582	HIT
protocatechuic acid	PRKCZ	5590	HIT
procyanidin	CDK1	983	STITCH
procyanidin	FOXO1	2308	STITCH
Pimaric Acid?	FCER1G	2207	STITCH
Pimaric Acid?	KCNMA1	3778	STITCH
Pimaric Acid?	KCNMB1	3779	STITCH
Pimaric Acid?	SART1	9092	STITCH
p-cymene（thymol）	ELANE	1991	HIT
panaxadiol,panaxatrio1,oleanolic acid,3-epioleanolic acid,cinnzeylanine,cinncassiols A,cinncassiols B	NR3C1	2908	HIT,STITCH
panaxadiol,panaxatrio1,Ginsenoside Rg3,bufalin,Vanillin	MMP9	4318	HIT,STITCH
palmitic acid	IL10	3586	HIT
palmitic acid	PCYT1A	5130	HIT
palmitic acid	PTEN	5728	HIT
palmitic acid	SLC22A5	6584	HIT
palmitic acid	TEP1	7011	HIT
oleanonic	GPBAR1	151306	STITCH

acid,Normuscone,3-methylcyclotridecan-2-one,cyclotetradecanone,oleanolic acid,Cholic acid,3-epioleanolic acid			
oleanolic acid,Ginsenoside Rf(Dammarane,b-D-glucopyranoside deriv),Ginsenoside Rg3,Ginsenoside Rh2,Deoxycholic acid,3-epioleanolic acid,linalool,cinnamaldehyde	PTGS2	5743	STITCH,HIT,HIT
oleanolic acid,Ginsenoside Fc,Ginsenoside Ra1,Ginsenoside Ra2,Ginsenoside Ra3,Ginsenoside Rb2(20-[(6-O-alpha-L-arabinopyranosyl-beta-D-glucopyranosyl)oxy]-12beta-hydroxydammar-24-en-3beta-yl 2-O-beta-D-glucopyranosyl-beta-D-glucopyranoside),Ginsenoside Rb3(Dammarane,b-D-glucopyranoside deriv),Ginsenoside Rs1,Ginsenoside Rs2,Notoginsenoside Fa,Quinquenoside R1,3-epioleanolic acid,cinnamaldehyde,cinnamoside	NFE2L2	4780	STITCH,HIT
oleanolic acid,5-Hydroxyindoleacetic acid (5-HIAA)(5-Hydroxyindole-3-acetic Acid),3-epioleanolic acid	TOP1	7150	STITCH
oleanolic acid,3-epioleanolic acid,epoxycinnamyl cinnamate,cinnamtannin A2,cinnamtannin A3,（procyanidin B-2 6-C-β-D-glucopyranoside）,cassioside	TOP2A	7153	STITCH
oleanolic acid,3-epioleanolic acid,dehydroabietic acid,linoleic acid	PPARA	5465	STITCH
oleanolic acid,3-epioleanolic acid	CASP8	841	STITCH
oleanolic acid,3-epioleanolic acid	MAPK14	1432	STITCH
oleanolic acid,3-epioleanolic acid	PTGIR	5739	STITCH
oleanolic acid,3-epioleanolic acid	PTGIS	5740	STITCH
oleanolic acid,3-epioleanolic acid	PTPN1	5770	STITCH
oleanolic acid,3-epioleanolic acid	AKR1B10	57016	STITCH
Notoginsenoside R1	SELE	6401	HIT
Normuscone,3-methylcyclotridecan-3-one,cyclotetradecanone,Testosterone	AR	367	STITCH
Normuscone,3-methylcyclotridecan-1-one,cyclotetradecanone	HACL1	26061	STITCH
nicotinamide,cinncassiols C3	PARP1	142	STITCH

nicotinamide,adenine,uracil	PNP	4860	STITCH
nicotinamide	PARP4	143	STITCH
nicotinamide	ART3	419	STITCH
nicotinamide	BST1	683	STITCH
nicotinamide	CD38	952	STITCH
nicotinamide	NNMT	4837	STITCH
nicotinamide	PARP3	10039	STITCH
nicotinamide	NAMPT	10135	STITCH
nicotinamide	SIRT5	23408	STITCH
nicotinamide	SIRT1	23411	STITCH
nicotinamide	SIRT6	51548	STITCH
nicotinamide	NMNAT1	64802	STITCH
nicotinamide	TNKS2	80351	STITCH
nicotinamide	PARP9	83666	STITCH
nicotinamide	NAPRT1	93100	STITCH
nicotinamide	ART5	116969	STITCH
nicotinamide	PARP15	165631	STITCH
nicotinamide	NMNAT3	349565	STITCH
myrcene	ATP6V1E1	529	STITCH
myrcene	HOXA9	3205	STITCH
myrcene	HOXD9	3235	STITCH
myrcene	IPP	3652	STITCH
myrcene	RPS3A	6189	STITCH
myrcene	ZNF224	7767	STITCH
myrcene	GGPS1	9453	STITCH
myrcene	PDSS1	23590	STITCH
myrcene	SAGE1	55511	STITCH
myrcene	PDSS2	57107	STITCH
myrcene	SDR16C5	195814	STITCH
Muscopyridine,Hydroxymuscopyridine A,Hydroxymuscopyridine B,beta-pinene	CNGA2	1260	STITCH
Muscopyridine,Hydroxymuscopyridine A,Hydroxymuscopyridine B,beta-pinene	CNGA3	1261	STITCH
Muscopyridine,Hydroxymuscopyridine A,Hydroxymuscopyridine B	OR8D1	283159	STITCH
Muscopyran,Testosterone	CYP17A1	1586	STITCH
Muscopyran,Cholic acid,benzoic acid	CES1	1066	STITCH
Muscol,estradiol,oleanolic acid,3-epioleanolic acid	CYP1A2	1544	STITCH
Muscol,Deoxycholic acid	VCAM1	7412	STITCH,HIT
Muscol,Butanoic acid,4-terpineol	BCHE	590	STITCH
Muscol	ACHE	43	STITCH
Muscol	CD63	967	STITCH

Muscol	CFTR	1080	STITCH
Muscol	CYP2B6	1555	STITCH
Muscol	CYP2C19	1557	STITCH
Muscol	CYP2E1	1571	STITCH
Methyl 4-hydroxycinnamate(methyl p-couma)	AKR1B1	231	STITCH
marinobufagenin,Marinobufagin	ATP4B	496	STITCH
marinobufagenin,Marinobufagin	CTSS	1520	STITCH
marinobufagenin,Marinobufagin	DNAH8	1769	STITCH
marinobufagenin,Marinobufagin	ERG	2078	STITCH
marinobufagenin,Marinobufagin	FGF10	2255	STITCH
marinobufagenin,Marinobufagin	FLI1	2313	STITCH
marinobufagenin,Marinobufagin	GNB3	2784	STITCH
marinobufagenin,Marinobufagin	REN	5972	STITCH
marinobufagenin,Marinobufagin	SRC	6714	STITCH
marinobufagenin,Marinobufagin	ATP11A	23250	STITCH
lyoniresinol-3α-o-β-D-glucopyranoside	MAP2K1	5604	STITCH
linoleic acid	ALOX15	246	STITCH
linoleic acid	ALOX15B	247	STITCH
linoleic acid	ACSL1	2180	STITCH
linoleic acid	GPX2	2877	STITCH
linoleic acid	FADS1	3992	STITCH
linoleic acid	PLA2G1B	5319	STITCH
linoleic acid	FADS2	9415	STITCH
linoleic acid	ELOVL2	54898	STITCH
linoleic acid	ELOVL5	60481	STITCH
linoleic acid	ACSBG2	81616	STITCH
linoleic acid	PLA2G12B	84647	STITCH
linoleic acid	PLA2G4D	283748	STITCH
linalool	ADORA2A	135	HIT
linalool	KATNA1	11104	HIT
limonene	BAD	572	HIT
leucine	DROSHA	29102	STITCH
L-cis-Diltiazem HCl	CNGA1	1259	STITCH
l-benzoyl-3-phenylpropyne	NUCB1	4924	STITCH
l，8-cineole	IL5	3567	HIT
l，8-cineole	PRODH	5625	HIT
Isopimaric acid	PTGER2	5732	STITCH
isoborneol	SLC7A1	6541	STITCH
isoborneol	SLC7A2	6542	STITCH
isoborneol	SLC7A4	6545	STITCH
isoborneol	DICER1	23405	STITCH
isoborneol	SLC7A14	57709	STITCH

isoborneol	SLC7A3	84889	STITCH
Hyodeoxycholic acid,bilirubin	UGT2B4	7363	STITCH
Hyodeoxycholic acid	GHDC	84514	STITCH
Glycocholic acid,cinncassiols D4	CLPS	1208	STITCH
Glycocholic acid	BAAT	570	STITCH
Glycocholic acid	CEL	1056	STITCH
Glycocholic acid	ODC1	4953	STITCH
Glycocholic acid	PNLIP	5406	STITCH
Glycocholic acid	SCP2	6342	STITCH
Glycocholic acid	SLCO1A2	6579	STITCH
Glycocholic acid	ABCC3	8714	STITCH
Glycocholic acid	SLCO1B1	10599	STITCH
Glycocholic acid	SLCO1B3	28234	STITCH
Ginsenoside Ro	AKTIP	64400	STITCH
Ginsenoside Rh2,cinnamaldehyde	NFKBIA	4792	HIT,HIT
Ginsenoside Rh2	ADCYAP1	116	HIT
Ginsenoside Rh2	CASP1	834	HIT
Ginsenoside Rh2	MAP2K4	6416	HIT
Ginsenoside Rh2	SLC2A4	6517	HIT
Ginsenoside Rh2	PSMG1	8624	HIT
Ginsenoside Rh1,Ginsenoside Rh2	BCL2A1	597	STITCH
Ginsenoside Rh1,Ginsenoside Rh2	RHAG	6005	STITCH
Ginsenoside Rg3,5-Hydroxyindoleacetic acid (5-HIAA)(5-Hydroxyindole-3-acetic Acid)	PPARG	5468	HIT,STITCH
Ginsenoside Rg3	HTR3A	3359	HIT
Ginsenoside Rg3	PRKAG2	51422	HIT
Ginsenoside Rg2,Notoginsenoside R2	GSK3B	2932	STITCH
Ginsenoside Rg2,Notoginsenoside R2	STK11	6794	STITCH
Ginsenoside Rg2,Notoginsenoside R2	NR0B2	8431	STITCH
Ginsenoside Rg1,protocatechuic acid	PRKCB	5579	HIT,HIT
Ginsenoside Rg1,palmitic acid	COL1A1	1277	HIT,HIT
Ginsenoside Rg1,Butanoic acid,(-)-epicatechin	IL2	3558	HIT,STITCH,HIT
Ginsenoside Rg1	ACTA2	59	HIT
ginsenoside rg1	ADRB2	154	HIT
Ginsenoside Rg1	CDH1	999	HIT
Ginsenoside Rg1	FN1	2335	HIT
Ginsenoside Rg1	SMAD2	4087	HIT
Ginsenoside Rg1	TGFB1	7040	HIT
Ginsenoside Rg1	THBS1	7057	HIT
Ginsenoside	IFNG	3458	HIT,HIT

Rf(Dammarane,b-D-glucopyranoside deriv),Ginsenoside Rg3,Ginsenoside Rh2,limonene			
Ginsenoside Rf(Dammarane,b-D-glucopyranoside deriv),Ginsenoside Rg3,Ginsenoside Rh2,l，8-cineole,palmitic acid,(-)-epicatechin	TNF	7124	HIT,HIT
Ginsenoside Rf(Dammarane,b-D-glucopyranoside deriv),Ginsenoside Rg3,Ginsenoside Rh2,l，8-cineole	IL1B	3553	HIT,HIT
Ginsenoside Rf(Dammarane,b-D-glucopyranoside deriv),Ginsenoside Rg1,Ginsenoside Rg3,limonene,l，8-cineole	IL4	3565	HIT,HIT
Ginsenoside Re,oleanolic acid,Ginsenoside Rd,Ginsenoside Rh2,bufalin,Butanoic acid,3-epioleanolic acid,(-)-epicatechin,cinncassiols C3	CASP3	836	HIT,STITCH,HIT
Ginsenoside Re,Ginsenoside Rg3,Ginsenoside Rh2,linalool	NOS2	4843	HIT,HIT
Ginsenoside Re	NOS3	4846	HIT
Ginsenoside Rd,Ginsenoside Re,Ursodeoxycholic acid,limonene,palmitic acid	BCL2	596	HIT,HIT
Ginsenoside Rd,Ginsenoside Re,Ginsenoside Rh2,bufalin,cinncassiols C3	BAX	581	HIT,STITCH
Ginsenoside Rd	PSMD3	5709	HIT
Ginsenoside Rc,Ginsenoside Re	FOS	2353	HIT
Ginsenoside Rc	CYP2C9	1559	HIT
Ginsenoside Rb1,Ginsenoside Rg1	AHR	196	HIT
ethylcinnamate	PDCL2	132954	STITCH
estradiol,Ginsenoside Rg1,Bufoserotonins A,Bufoserotonins B,Benzyl cinnamate	CYP1A1	1543	STITCH,HIT
estradiol,Ginsenoside Rb1	ESR2	2100	STITCH,HIT
estradiol,Bufoserotonins B,Abietatriene-3β-ol,4-ethyphenol	ESR1	2099	STITCH
estradiol,Abietatriene-3β-ol	HSD17B1	3292	STITCH
estradiol,?5-Hydroxymethyl-2-furaldehyde	SULT1A1	6817	STITCH

estradiol	GLUL	2752	STITCH
estradiol	OXTR	5021	STITCH
estradiol	ABCB1	5243	STITCH
estradiol	SULT1E1	6783	STITCH
estradiol	TFF1	7031	STITCH
estradiol	NCOA3	8202	STITCH
estradiol	HSD17B7	51478	STITCH
dihydrocoumarone	PON1	5444	STITCH
dihydrocoumarone	PON2	5445	STITCH
dihydrocoumarone	PON3	5446	STITCH
desacetylbufotalin	POLI	11201	STITCH
Deoxycholic acid,procyanidin,cinncassiols C3	TP53	7157	HIT,STITCH
Deoxycholic acid,limonene	HMGCR	3156	HIT,HIT
Deoxycholic acid	BIRC2	329	HIT
Deoxycholic acid	BAK1	578	HIT
Deoxycholic acid	EGFR	1956	HIT
Deoxycholic acid	GPT	2875	HIT
Deoxycholic acid	ICAM1	3383	HIT
Deoxycholic acid	L1CAM	3897	HIT
Deoxycholic acid	MCL1	4170	HIT
Deoxycholic acid	MUC2	4583	HIT
Deoxycholic acid	PTPRG	5793	HIT
Deoxycholic acid	BAG3	9531	HIT
D-borneol	OXT	5020	STITCH
D-borneol	PPA1	5464	STITCH
D-borneol	RB1	5925	STITCH
D-borneol	ULK1	8408	STITCH
D-borneol	CAMKK2	10645	STITCH
D-borneol	CAMKK1	84254	STITCH
coumarin	CYP2A13	1553	STITCH
cinobufagin,Muscone,Muscol,Testosterone,oleanolic acid,Ginsenoside Rf(Dammarane,b-D-glucopyranoside deriv),bufalin,Ursodeoxycholic acid,Hyodeoxycholic acid,3-epioleanolic acid	CYP3A4	1576	STITCH,HIT
cinobufagin 3-acetate,1-hydroxy-cinobufagin,Cinobufotalin,19-oxo-Cinobufotalin	GLRA1	2741	STITCH
cinobufagin 3-acetate,1-hydroxy-cinobufagin,Cinobufotalin,19-oxo-Cinobufotalin	RRM2	6241	STITCH

cinnzeylanine,cinncassiols D1	ACADL	33	STITCH
cinnzeylanine,cinncassiols D1	TBL1X	6907	STITCH
cinnzeylanine,cinncassiols D1	CDK5RAP1	51654	STITCH
cinnzeylanine,cinncassiols D1	EXOSC4	54512	STITCH
cinnzeylanine,cinncassiols D1	IFT80	57560	STITCH
cinnzeylanine,cinncassiols D1	TBL1XR1	79718	STITCH
cinnzeylanine,cinncassiols D1	TBL1Y	90665	STITCH
cinnzeylanine,cinncassiols D1	WDR17	116966	STITCH
cinnzeylanine,cinncassiols D1	EXOSC6	118460	STITCH
cinnzeylanine,cinncassiols A,cinncassiols B	ADCY1	107	STITCH
cinncassiols,cinncassiols D3	MT1X	4501	STITCH
cinncassiols,cinncassiols D3	DOCK4	9732	STITCH
cinncassiols D4	AGA	175	STITCH
cinncassiols D4	CHIT1	1118	STITCH
cinncassiols D4	HEXA	3073	STITCH
cinncassiols D4	HEXB	3074	STITCH
cinncassiols D4	RAB27A	5873	STITCH
cinncassiols D4	SI	6476	STITCH
cinncassiols D4	RPL23	9349	STITCH
cinncassiols D4	SF3B4	10262	STITCH
cinncassiols D4	CHIA	27159	STITCH
cinncassiols D4	SIAE	54414	STITCH
cinncassiols D4	TASP1	55617	STITCH
cinncassiols D4	ASRGL1	80150	STITCH
cinncassiols D4	OVCA2	124641	STITCH
cinncassiols D4	ASPG	374569	STITCH
cinncassiols C3	CCND1	595	STITCH
Cinnamyl acetate,myrcene,cinnamyl acetate	TKT	7086	STITCH
Cinnamyl acetate,myrcene,cinnamyl acetate	TKTL1	8277	STITCH
Cinnamyl acetate,myrcene,cinnamyl acetate	TKTL2	84076	STITCH
Cinnamyl acetate,cinnamyl acetate	CPT1A	1374	STITCH
Cinnamyl acetate,cinnamyl acetate	CPT1B	1375	STITCH
Cinnamyl acetate,cinnamyl acetate	ARL5A	26225	STITCH
Cinnamyl acetate,cinnamyl acetate	CPT1C	126129	STITCH
cinnamaldehyde	C5AR1	728	HIT
cinnamaldehyde	IFNB1	3456	HIT
cinnamaldehyde	IRF3	3661	HIT
cinnamaldehyde	RELA	5970	HIT
cinnamaldehyde	TLR4	7099	HIT

cinnamaldehyde	TXNRD1	7296	HIT
cinnamaldehyde	TRPV1	7442	HIT
cinnamaldehyde	TRPV4	59341	HIT
Cholic acid,Glycocholic acid	SLC10A1	6554	STITCH
Cholic acid,Glycocholic acid	SLC10A2	6555	STITCH
Cholic acid,Glycocholic acid	ABCB11	8647	STITCH
Cholic acid,Glycocholic acid	SLC27A5	10998	STITCH
Cholic acid,Chenodeoxycholic acid,Glycocholic acid	NR1H4	9971	STITCH,HIT
Cholic acid	COX5B	1329	STITCH
Cholic acid	COX6A2	1339	STITCH
Cholic acid	COX6B1	1340	STITCH
Cholic acid	COX7A1	1346	STITCH
Cholic acid	COX7C	1350	STITCH
Cholic acid	ESRRG	2104	STITCH
Cholic acid	FECH	2235	STITCH
Cholic acid	COX5A	9377	STITCH
Cholesterol,Notoginsenoside R2	SREBF2	6721	STITCH
Cholesterol,Glycocholic acid	ALB	213	STITCH
Cholesterol,Deoxycholic acid	LDLR	3949	STITCH,HIT
Cholesterol	ABCA1	19	STITCH
Cholesterol	APOA1	335	STITCH
Cholesterol	APOB	338	STITCH
Cholesterol	APOE	348	STITCH
Cholesterol	CAV1	857	STITCH
Cholesterol	CETP	1071	STITCH
Cholesterol	CYP11A1	1583	STITCH
Cholesterol	CYP27A1	1593	STITCH
Cholesterol	LCAT	3931	STITCH
Cholesterol	LPA	4018	STITCH
Cholesterol	LPL	4023	STITCH
Cholesterol	SOAT1	6646	STITCH
Cholesterol	SCAP	22937	STITCH
Cholesterol	ABCG8	64241	STITCH
cholest-4-en-4-one,Testosterone	AKR1D1	6718	STITCH
cholest-4-en-3-one ,Cholesterol,Cholic acid,Deoxycholic acid	CYP7A1	1581	STITCH,HIT
cassioside	FDFT1	2222	STITCH
cassioside	LSS	4047	STITCH
cassioside	RPE65	6121	STITCH
cassioside	BCMO1	53630	STITCH
cassioside	BCO2	83875	STITCH
cassioside	ZNF644	84146	STITCH

cassioside	ISPD	729920	STITCH
camphene	CNR1	1268	STITCH
calamenene	HTR1A	3350	STITCH
Butanoic acid	CCK	885	STITCH
Butanoic acid	GAST	2520	STITCH
Butanoic acid	GCG	2641	STITCH
Butanoic acid	GCGR	2642	STITCH
Butanoic acid	FFAR1	2864	STITCH
Butanoic acid	FFAR2	2867	STITCH
Butanoic acid	GSN	2934	STITCH
Butanoic acid	NTS	4922	STITCH
Butanoic acid	PTAFR	5724	STITCH
Butanoic acid	TAC1	6863	STITCH
Butanoic acid	TACR2	6865	STITCH
Butanoic acid	TACR1	6869	STITCH
Butanoic acid	TACR3	6870	STITCH
Butanoic acid	TRH	7200	STITCH
Butanoic acid	LPAR2	9170	STITCH
Butanoic acid	NPS	594857	STITCH
Bufoserotonins B,5-Hydroxyindoleacetic acid (5-HIAA)(5-Hydroxyindole-3-acetic Acid)	SPR	6697	STITCH
Bufoserotonins A,Bufoserotonins B,5-Hydroxyindoleacetic acid (5-HIAA)(5-Hydroxyindole-3-acetic Acid)	ASMT	438	STITCH
Bufoserotonins A,Bufoserotonins B	AANAT	15	STITCH
Bufoserotonins A,Bufoserotonins B	IDO1	3620	STITCH
Bufoserotonins A,Bufoserotonins B	MTNR1A	4543	STITCH
Bufoserotonins A,Bufoserotonins B	MTNR1B	4544	STITCH
Bufoserotonins A,Bufoserotonins B	NQO2	4835	STITCH
Bufoserotonins A	HTR1D	3352	STITCH
Bufoserotonins A	HTR2A	3356	STITCH
bufalin,cinnamtannin A2,cinnamtannin A3,（procyanidin B-2 6-C-β-D-glucopyranoside）	CASP9	842	STITCH
bufalin,cinnamtannin A2,cinnamtannin A3,（procyanidin B-2 6-C-β-D-glucopyranoside）	MAPK1	5594	STITCH
bufalin,cinnamtannin A2,cinnamtannin A3,（procyanidin B-2 6-C-β-D-glucopyranoside）	MAPK3	5595	STITCH

bufalin	GNRH1	2796	STITCH
bufalin	MAPK8	5599	STITCH
bufalin	MAPK9	5601	STITCH
bufalin	TF	7018	STITCH
bufalin	EIF2AK3	9451	STITCH
bufalin	ZNF263	10127	STITCH
bilirubin,Vanillin,Vanillic acid,β-phenylpropionic acid	UGT1A10	54575	STITCH
bilirubin,Vanillin,Vanillic acid,β-phenylpropionic acid	UGT1A8	54576	STITCH
bilirubin,Vanillin,Vanillic acid,β-phenylpropionic acid	UGT1A7	54577	STITCH
bilirubin,D-borneol	UGT1A5	54579	STITCH
bilirubin,D-borneol	UGT1A9	54600	STITCH
bilirubin,D-borneol	UGT1A4	54657	STITCH
bilirubin,D-borneol	UGT1A3	54659	STITCH
bilirubin,coumarin	UGT2B15	7366	STITCH
bilirubin	ALPL	249	STITCH
bilirubin	ALPP	250	STITCH
bilirubin	ALPPL2	251	STITCH
bilirubin	BLVRA	644	STITCH
bilirubin	HMOX1	3162	STITCH
bilirubin	HMOX2	3163	STITCH
bilirubin	UGT2B7	7364	STITCH
bilirubin	UGT2B10	7365	STITCH
bilirubin	UGT2B11	10720	STITCH
beta-pinene,myrcene	COLQ	8292	STITCH
beta-pinene,myrcene	SPEN	23013	STITCH
beta-pinene,myrcene	TPSD1	23430	STITCH
beta-pinene	ALAS1	211	STITCH
beta-pinene	ST3GAL3	6487	STITCH
Benzyl cinnamate	AKR1C2	1646	STITCH
Benzyl cinnamate	NQO1	1728	STITCH
Benzyl cinnamate	MT1A	4489	STITCH
Benzyl cinnamate	MT2A	4502	STITCH
Benzyl cinnamate	TXN	7295	STITCH
Benzyl cinnamate	MAFF	23764	STITCH
Benzyl cinnamate	FAM60A	58516	STITCH
benzoic acid	RAB9A	9367	STITCH
benzoic acid	HRSP12	10247	STITCH
benzoic acid	GLYAT	10249	STITCH
benzoic acid	PRDX5	25824	STITCH
benzoic acid	ACSM1	116285	STITCH

benzoic acid	ACSM2B	348158	STITCH
benzaldehyde	ALDH1A2	8854	STITCH
benzaldehyde	ALDH8A1	64577	STITCH
a-terpineol,(-)-epicatechin	IL6	3569	HIT
arenobufagin,Bufarenogin,Ψ-bufarenogin （psi-bufarenogin),telocinobufagin,Bufotalidin(Hellebrigenin)	SDF4	51150	STITCH
alpha-pinene	LHFPL5	222662	STITCH
adenine,uracil	SLC29A2	3177	STITCH
adenine,cassioside	SLC29A3	55315	STITCH
adenine	ACACB	32	STITCH
adenine	ACP1	52	STITCH
adenine	ACVR2B	93	STITCH
adenine	ADA	100	STITCH
adenine	APRT	353	STITCH
adenine	HSP90AA1	3320	STITCH
adenine	MTAP	4507	STITCH
adenine	PNMT	5409	STITCH
adenine	PYGM	5837	STITCH
adenine	SRPK2	6733	STITCH
adenine	STK24	8428	STITCH
adenine	SF3B1	23451	STITCH
adenine	SF3B14	51639	STITCH
adenine	CHFR	55743	STITCH
adenine	PECR	55825	STITCH
adenine	MRI1	84245	STITCH
adenine	ADAT3	113179	STITCH
Abietatriene-3β-ol	STS	412	STITCH
Abietatriene-3β-ol	RXRA	6256	STITCH
5-Hydroxyindoleacetic acid (5-HIAA)(5-Hydroxyindole-3-acetic Acid),calamenene	TH	7054	STITCH
5-Hydroxyindoleacetic acid (5-HIAA)(5-Hydroxyindole-3-acetic Acid)	ALDH2	217	STITCH
5-Hydroxyindoleacetic acid (5-HIAA)(5-Hydroxyindole-3-acetic Acid)	ALDH1B1	219	STITCH
5-Hydroxyindoleacetic acid (5-HIAA)(5-Hydroxyindole-3-acetic Acid)	ALDH9A1	223	STITCH
5-Hydroxyindoleacetic acid	ALDH3A2	224	STITCH

(5-HIAA)(5-Hydroxyindole-3-acetic Acid)			
5-Hydroxyindoleacetic acid (5-HIAA)(5-Hydroxyindole-3-acetic Acid)	AOX1	316	STITCH
5-Hydroxyindoleacetic acid (5-HIAA)(5-Hydroxyindole-3-acetic Acid)	CBR1	873	STITCH
5-Hydroxyindoleacetic acid (5-HIAA)(5-Hydroxyindole-3-acetic Acid)	DDC	1644	STITCH
5-Hydroxyindoleacetic acid (5-HIAA)(5-Hydroxyindole-3-acetic Acid)	GCH1	2643	STITCH
5-Hydroxyindoleacetic acid (5-HIAA)(5-Hydroxyindole-3-acetic Acid)	MAOA	4128	STITCH
5-Hydroxyindoleacetic acid (5-HIAA)(5-Hydroxyindole-3-acetic Acid)	MAOB	4129	STITCH
5-Hydroxyindoleacetic acid (5-HIAA)(5-Hydroxyindole-3-acetic Acid)	SLC6A4	6532	STITCH
5-Hydroxyindoleacetic acid (5-HIAA)(5-Hydroxyindole-3-acetic Acid)	TPH1	7166	STITCH
4-ethyphenol	GPATCH1	55094	STITCH
3-ethyphenol,β-phenylpropionic acid	TYR	7299	STITCH
3'-○-methyl-(-)-epicatechin,5,3'-di-O-methylate-(-)-epicatechin,5,7,3'-tri-O-methylate-(-)-epicatechin,〔4'-○-methyl-(+)-catechin〕,（7,4'-di-O-methylate-(+)-catechin），（5,7,4'-tri-O-methylate-(+)-catechin），5，7-dimethyl-3'，4'-di-○-methylene-(±)-epicatechin	CA3	761	STITCH
3'-○-methyl-(-)-epicatechin,5,3'-di-O-methylate-(-)-epicatechin,5,7,3'-tri-O-methylate-(-)-epicatechin,〔4'-○-methyl-(+)-catechin〕,（7,4'-di-O-methylate-(+)-catechin），（5,7,4'-tri-O-methylate-(+)-catechin），5，7-dimethyl-3'，4'-di-○-methylene-(±)-epicatechin	CA4	762	STITCH

3'-○-methyl-(-)-epicatechin,5,3'-di-O-methylate-(-)-epicatechin,5,7,3'-tri-O-methylate-(-)-epicatechin,〔4'-○-methyl-(+)-catechin〕,（7,4'-di-O-methylate-(+)-catechin）,（5,7,4'-tri-O-methylate-(+)-catechin）,5，7-dimethyl-3'，4'-di-○-methylene-(±)-epicatechin	CA5A	763	STITCH
3'-○-methyl-(-)-epicatechin,5,3'-di-O-methylate-(-)-epicatechin,5,7,3'-tri-O-methylate-(-)-epicatechin,〔4'-○-methyl-(+)-catechin〕,（7,4'-di-O-methylate-(+)-catechin）,（5,7,4'-tri-O-methylate-(+)-catechin）,5，7-dimethyl-3'，4'-di-○-methylene-(±)-epicatechin	CA6	765	STITCH
3'-○-methyl-(-)-epicatechin,5,3'-di-O-methylate-(-)-epicatechin,5,7,3'-tri-O-methylate-(-)-epicatechin,〔4'-○-methyl-(+)-catechin〕,（7,4'-di-O-methylate-(+)-catechin）,（5,7,4'-tri-O-methylate-(+)-catechin）,5，7-dimethyl-3'，4'-di-○-methylene-(±)-epicatechin	CA7	766	STITCH
3'-○-methyl-(-)-epicatechin,5,3'-di-O-methylate-(-)-epicatechin,5,7,3'-tri-O-methylate-(-)-epicatechin,〔4'-○-methyl-(+)-catechin〕,（7,4'-di-O-methylate-(+)-catechin）,（5,7,4'-tri-O-methylate-(+)-catechin）,5，7-dimethyl-3'，4'-di-○-methylene-(±)-epicatechin	CA9	768	STITCH
3'-○-methyl-(-)-epicatechin,5,3'-di-O-methylate-(-)-epicatechin,5,7,3'-tri-O-methylate-(-)-epicatechin,〔4'-○-methyl-(+)-catechin〕,（7,4'-di-O-methylate-(+)-catechin）,（5,7,4'-tri-O-methylate-(+)-catechin）,5，7-dimethyl-3'，4'-di-○-methylene-(±)-epicatechin	CA12	771	STITCH
3'-○-methyl-(-)-epicatechin,5,3'-di-O-methylate-(-)-epicatechin,5,7,3'-tri-O-methylate-(-)-epicatechin,〔4'-○-methyl-(+)-catechin〕,	CA5B	11238	STITCH

（7,4'-di-O-methylate-(+)-catechin），（5,7,4'-tri-O-methylate-(+)-catechin），5，7-dimethyl-3'，4'-di-○-methylene-(±)-epicatechin			
3，4，5-trimethoxyphenol-β-D-apiofuranosyl(1→6)-β-D-glucopyranoside	AMY2A	279	STITCH
3，4，5-trimethoxyphenol-β-D-apiofuranosyl(1→6)-β-D-glucopyranoside	GANC	2595	STITCH
3，4，5-trimethoxyphenol-β-D-apiofuranosyl(1→6)-β-D-glucopyranoside	TGM4	7047	STITCH
3，4，5-trimethoxyphenol-β-D-apiofuranosyl(1→6)-β-D-glucopyranoside	GANAB	23193	STITCH
2-piperidinecarboxylic acid,benzoic acid	DAO	1610	STITCH
2-piperidinecarboxylic acid,5-Hydroxyindoleacetic acid (5-HIAA)(5-Hydroxyindole-3-acetic Acid)	ALDH7A1	501	STITCH
2-piperidinecarboxylic acid	ATP5A1	498	STITCH
2-piperidinecarboxylic acid	ATP5B	506	STITCH
2-piperidinecarboxylic acid	ATP5C1	509	STITCH
2-piperidinecarboxylic acid	CAT	847	STITCH
2-piperidinecarboxylic acid	F2	2147	STITCH
2-piperidinecarboxylic acid	PEX1	5189	STITCH
2-piperidinecarboxylic acid	PHYH	5264	STITCH
2-piperidinecarboxylic acid	PIN1	5300	STITCH
2-piperidinecarboxylic acid	PREP	5550	STITCH
2-piperidinecarboxylic acid	CLPP	8192	STITCH
2-piperidinecarboxylic acid	GNPAT	8443	STITCH
2-piperidinecarboxylic acid	PREPL	9581	STITCH
2-piperidinecarboxylic acid	AASS	10157	STITCH
2-piperidinecarboxylic acid	PIPOX	51268	STITCH
2-piperidinecarboxylic acid	PEX26	55670	STITCH
2-ethyphenol	FTL	2512	STITCH
?benzyl benzoate（Ascabin）,benzyl benzoate	LIPE	3991	STITCH
?5-Hydroxymethyl-2-furaldehyde	DCX	1641	STITCH
?5-Hydroxymethyl-2-furaldehyde	HBA2	3040	STITCH
?5-Hydroxymethyl-2-furaldehyde	HBB	3043	STITCH
?5-Hydroxymethyl-2-furaldehyde	SULT1A2	6799	STITCH

（?(-)-epicatechin 3-O-β-D-glucopyranoside）	RPL8	6132	STITCH
（?(-)-epicatechin 3-O-β-D-glucopyranoside）	SLC5A1	6523	STITCH
（?(-)-epicatechin 3-O-β-D-glucopyranoside）	TBP	6908	STITCH
（?(-)-epicatechin 3-O-β-D-glucopyranoside）	TBPL1	9519	STITCH
（?(-)-epicatechin 3-O-β-D-glucopyranoside）	MRPL2	51069	STITCH
（?(-)-epicatechin 3-O-β-D-glucopyranoside）	TBPL2	387332	STITCH
(-)-epicatechin	COMT	1312	HIT
(-)-epicatechin	CREB1	1385	HIT
(-)-epicatechin	ACE	1636	HIT
(-)-epicatechin	GCLC	2729	HIT
(-)-epicatechin	GRIA2	2891	HIT
(-)-epicatechin	GRIN1	2902	HIT
(-)-epicatechin	GSS	2937	HIT
(-)-epicatechin	HAS2	3037	HIT
(-)-epicatechin	IL1A	3552	HIT
(-)-epicatechin	PLAT	5327	HIT
(-)-epicatechin	PLAU	5328	HIT
(-)-epicatechin	POR	5447	HIT
(-)-epicatechin	CCL2	6347	HIT
(-)-epicatechin	DUOX2	50506	HIT
(-)-epicatechin	CRTC2	200186	HIT
（(-)-epicatechin 8-C-β-D-glucopyranoside ）	MANBA	4126	STITCH
（ (-)-epicatechin 6-C-β-D-glucopyranoside ）	SLC5A2	6524	STITCH

Table S4. SBP's plasma absorbed compounds and their targets

Component	Medicinal materials	Target	Database
Muscone	Moschus	CYP3A4	STITCH
Ginsenoside Rb1	Total ginsenoside ginseng root	AHR	HIT
		BHLHE76	
		ESR2	
		ESTRB	
		NR3A2	
Ginsenoside Rb2(20-[(6-O-alpha-L-arabinopyran	Total ginsenoside ginseng root	NFE2L2	STITCH
		AKT1	

osyl-beta-D-glucopyranosyl)oxy]-12 beta-hydroxydammar-24-en-3beta-yl 2-O-beta-D-glucopyranosyl-beta-D-glucopyranoside)			
Ginsenoside Rb3(Dammarane,b-D-glucopyranoside deriv)	Total ginsenoside ginseng root	NFE2L2	STITCH
		AKT1	
Ginsenoside Rd	Total ginsenoside ginseng root	CASP3	HIT
		CPP32	
		PSMD3	
		BCL2	
		BCL2L4	
		BAX	
Ginsenoside Rc	Total ginsenoside ginseng root	FOS	HIT
		G0S7	
		CYP2C9	
		CYP2C10	
Ginsenoside Re	Total ginsenoside ginseng root	NOS3	HIT
		BAX	
		BCL2L4	
		NOS2	
		NOS2A	
		CASP3	
		CPP32	
		BCL2	
		FOS	
		G0S7	
Ginsenoside Rg1	Total ginsenoside ginseng root	AHR	HIT
		BHLHE76	
		CYP1A1	
		ACTA2	
		ACTSA	
		ACTVS	
		GIG46	
		TGFB1	
		TGFB	
		SMAD2	
		MADH2	
		MADR2	
		THBS1	
		TSP	
		TSP1	
		CDH1	

		CDHE	
		UVO	
		COL1A1	
		FN1	
		FN	
		PRKCB	
		PKCB	
		PRKCB1	
		IL2	
		IL4	
resibufogenin	Bufonis Venenum	PHKA1	STITCH
		ECEL1	
		TAF1	
		ZC3H15	
		DRG1	
		SERPINA6	
		RPS12	
		PGK1	
		RWDD1	
		GCN1L1	
gamabufotalin(Gamabufogenin)	Bufonis Venenum	PHKA1	STITCH
		ECEL1	
		TAF1	
		ZC3H15	
		DRG1	
		SERPINA6	
		RPS12	
		PGK1	
		RWDD1	
		GCN1L1	
arenobufagin	Bufonis Venenum	SDF4	STITCH
bufalin	Bufonis Venenum	CYP3A4	STITCH
		CASP3	
		BAX	
		CASP9	
		EIF2AK3	
		GNRH1	
		TF	
		ZNF263	
		MMP9	
		MAPK8	
		MAPK9	
		MAPK3	

		MAPK1	
1β-Hydroxybufalin	Bufonis Venenum	PHKA1	STITCH
		ECEL1	
		TAF1	
		ZC3H15	
		DRG1	
		SERPINA6	
		RPS12	
		PGK1	
		RWDD1	
		GCN1L1	
bufotalin	Bufonis Venenum	PHKA1	STITCH
		ECEL1	
		TAF1	
		ZC3H15	
		DRG1	
		SERPINA6	
		RPS12	
		PGK1	
		RWDD1	
		GCN1L1	
telocinobufagin	Bufonis Venenum	SDF4	STITCH
Cinobufotalin	Bufonis Venenum	RRM2	STITCH
		GLRA1	
Ursodeoxycholic acid	Bovis Calculus Artifactus	CYP3A4	HIT
		CYP3A3	
		FABP6	HIT
		ILBP	
		ILLBP	
		NCOA1	HIT
		BHLHE74	
		SRC1	
		BCL2	HIT
		E2F1	HIT
		RBBP3	
Cholic acid	Bovis Calculus Artifactus	ABCB11	STITCH
		FABP6	
		CES1	
		FECH	
		NR1H4	
		ESRRG	
		MT-CO2	
		COX5A	

		COX7A1	
		COX6A2	
		MT-CO3	
		MT-CO1	
		GPBAR1	
		COX5B	
		COX6B1	
		CYP7A1	
		SLC10A2	
		COX7C	
		SLC10A1	
		SLC27A5	
Deoxycholic acid	Bovis Calculus Artifactus	BAG3	HIT
		BIS	
		BIRC2	
		API1	
		IAP2	
		MIHB	
		RNF48	
		MCL1	
		BCL2L3	
		BAK1	
		BAK	
		BCL2L7	
		CDN1	
		TP53	
		P53	
		MUC2	
		SMUC	
		ICAM1	
		VCAM1	
		L1CAM	
		PTGS2	
		COX2	
		PTPRG	
		PTPG	
		EGFR	
		ERBB1	
		CYP7A1	
		CYP7	
		LDLR	
		GPT	
		AAT1	

		GPT1<br>HMGCR	
Chenodeoxycholic acid	Bovis Calculus Artifactus	NR1H4<br>BAR<br>FXR<br>HRR1<br>RIP14	HIT
Hyodeoxycholic acid	Bovis Calculus Artifactus	UGT2B4<br>CYP3A4<br>GHDC	STITCH
cinnamaldehyde	Styrax	C5AR1<br>C5AR<br>C5R1<br>TRPV1<br>VR1<br>TRPV4<br>VRL2<br>VROAC<br>NFKBIA<br>IKBA<br>MAD3<br>NFKBI<br>NFE2L2<br>NRF2<br>TXNRD1<br>GRIM12<br>KDRF<br>RELA<br>NFKB3<br>IRF3<br>TLR4<br>PTGS2<br>COX2<br>IFNB1<br>IFB<br>IFNB	HIT
cis-cinnamic acid	Styrax	CA1<br>CA2	STITCH
benzyl benzoate	Styrax	LIPE	STITCH
D-borneol	Borneolum Syntheticum	RB1<br>UGT1A5<br>UGT1A4<br>CAMKK2	STITCH

		CAMKK1	
		UGT1A3	
		UGT1A9	
		PPA1	
		UGT1A1	
		ULK1	
		OXT	
isoborneol	Borneolum Syntheticum	SLC7A2	STITCH
		SLC7A4	
		SLC7A1	
		ENSG00000188666	
		SLC7A3	
		SLC7A14	
		DICER1	
		RNASEN	

Table S5. Significant expressed genes and corresponding fold change values

Gene_Symbol	Entrez_Gene	foldchange
TRAF3IP3	80342	0.049808
GAD2	2572	18.57932
CYLC1	1538	18.10809
SYNGR1	9145	0.061548
NLGN4Y	22829	0.063076
MME	4311	15.64419
TACC1	6867	15.50599
TOX3	27324	15.42795
PRKDC	5591	0.065013
LCN2	3934	14.43118
ZKSCAN8	7745	0.069429
C3orf18	51161	0.06987
LAMA4	3910	0.073016
HEXA-AS1	80072	0.074715
LOXL2	4017	13.0397
RBMXL2	27288	13.02746
TNIP3	79931	0.078097
ELTD1	64123	12.73362
KRT83	3889	0.079213
TNFSF14	8740	0.079748
EPS15L1	58513	12.24958
PRX	57716	0.082222

ZC2HC1C	79696	0.084623
TEX13A	56157	11.79943
MBL2	4153	0.086092
ANXA13	312	11.44103
Igk	243469	0.088902
PELO	53918	0.088978
BAI3	577	0.089595
CFI	3426	0.093251
IGFBP7	3490	0.093929
CDH20	28316	0.094785
ECM2	1842	10.5127
SPAM1	6677	0.095205
EFCAB1	79645	0.095469
DIAPH3	81624	10.46563
SLC7A8	23428	10.42589
SAP30	8819	10.38012
PTPRT	11122	10.37189
DPEP3	64180	10.33414
CDH15	1013	10.28328
ATXN3	4287	0.097484
MUSK	4593	0.099133
XYLB	9942	0.10109
LAMP3	27074	0.101299
BHMT	635	9.857221
MCM3AP	8888	9.822597
ZNF135	7694	0.10245
MMP1	4312	9.705378
ISG20L2	81875	9.703212
OPRL1	4987	0.104977
DOCK5	80005	0.105559
CAMK1D	57118	0.106208
ANGPTL2	23452	0.106298
TTLL7	79739	0.106309
PTK2B	2185	0.107349
SEMA3D	223117	9.211773
GABRA5	2558	0.109001
ELF5	2001	9.153281
EPM2A	7957	0.109313
TLL1	7092	0.109628
ERV9-1	1.01E+08	9.060175
PPP1R3A	5506	0.110831
PARVB	29780	0.110934
TTC22	55001	0.111884

RASL12	51285	0.112133
ELL2	22936	8.895335
FGF20	26281	0.112481
C14orf105	55195	0.112763
DSC2	1824	8.817805
CIB2	10518	8.79141
MIR1257///TAF4	6874///100302168	8.785914
PRKG1	5592	0.113837
ENTPD3	956	8.776766
CASR	846	8.762664
TTTY2///TTTY2B	60439///100101117	8.760013
ADAM22	53616	0.114552
PCCA	5095	0.115014
TRPC6	7225	8.684741
UGT2B4	7363	8.669116
LZTS1	11178	0.115721
CD4	920	0.116837
AQP4	361	0.116923
CRHR1	1394	0.11697
FGB	2244	8.526688
PDLIM4	8572	0.11729
NFATC4	4776	0.117306
GABRG2	2566	0.117487
MSR1	4481	0.117944
CCIN	881	8.473448
FAM65B	9750	0.11856
CALD1	800	0.118984
GCM2	9247	8.381394
SPOCK3	50859	0.119771
SOCS1	8651	0.120055
CDKN1C	1028	8.310353
SIRPB1	10326	0.12066
IGH///IGHA1///IGHG1///IGHG2///IGHG3///IGHM	3492///3493///3500///3501///3502///3507	8.282598
ADH1B	125	0.120986
ARPP21	10777	0.122065
CAMP	820	0.12253
RECK	8434	8.156001
PLA2R1	22925	0.123234
SCG3	29106	0.123401

SPON1	10418	0.12396
HAL	3034	0.124515
CCDC134	79879	0.125059
SELPLG	6404	7.97903
LINC00965	349196	0.125383
PDPN	10630	0.127127
DYRK3	8444	0.127329
CCDC102B	79839	0.127625
IL5RA	3568	0.128061
TIAM2	26230	7.80199
TEK	7010	0.129225
PAK3	5063	0.129631
MST1L	11223	0.129675
LOC101060620///MAPK8IP1	9479///101060620	7.70517
PDLIM5	10611	0.130006
HGF	3082	7.691901
TNKS	8658	7.673146
TAZ	6901	0.130691
PCLO	27445	0.130696
CD69	969	7.629999
VSNL1	7447	7.629885
PTN	5764	7.629177
CWH43	80157	7.613785
PSMB9	5698	0.131491
ABCB11	8647	7.597977
DCX	1641	7.581249
TCF21	6943	7.553765
PTRF	284119	0.132907
ECRP	643332	7.508783
CDH6	1004	7.48641
CGA	1081	0.133665
CALY	50632	7.472534
C16orf70	80262	0.133868
ASTN1	460	7.46684
SMARCA4	6597	7.460428
IGHD	3495	7.406807
SMG7-AS1	284649	0.135044
DCHS2	54798	0.13511
CYP1B1	1545	7.394989
LOC100507388	1.01E+08	0.135247
LOC100996400///NOL4	8715///100996400	0.135313

EDA	1896	0.13577
PTPN20A///PTPN20B	26095///65312 9	0.137094
MAGI2	9863	0.137344
CASP1	834	7.280741
CCL23	6368	7.246165
SCGB1D1	10648	7.220643
CYP1A1	1543	7.197823
GPR98	84059	0.139598
DAB2	1601	0.139781
PTCD2	79810	0.139878
ADAM28	10863	0.139939
CCDC85B	11007	7.123477
ALB	213	0.140735
MYF5	4617	0.142132
ENG	2022	0.142194
IGF2BP3	10643	6.975194
PTGS2	5743	0.144067
CDKN2A	1029	0.144933
HBA1///HBA2	3039///3040	0.144972
DUSP1	1843	6.875058
PER2	8864	6.858962
CDKL5	6792	0.146056
IGHA1///IGHG1///IGHM///IGHV3-23///IGH V4-31	3493///3500/// 3507///28396// /28442	0.146131
EMX2	2018	0.146491
MORN1	79906	0.146525
GIMAP1-GIMAP5///GIMAP5	55340///10052 7949	6.815763
GPLD1	2822	6.783258
PCSK5	5125	6.767327
APOB	338	0.148074
BAIAP3	8938	0.148099
ANKRD2	26287	6.744425
SLC23A2	9962	0.148304
RTDR1	27156	0.148858
MAP2K2	5605	0.148968
SLC16A4	9122	6.709282
AURKC	6795	0.149083
BGN	633	0.149102
CMKLR1	1240	0.14968
LHCGR	3973	0.149763

GRIP1	23426	6.671348
APOBEC3F///APOBEC3G	60489///200316	0.150016
GYPA	2993	6.663177
CYP4F12	66002	6.642306
MAGEB4	4115	6.626046
EDNRA	1909	6.608504
BIN1	274	6.601771
KAT6A	7994	6.595417
LINC00939	400084	0.151836
SLC4A7	9497	6.584317
LOC100130331	1E+08	6.582577
SV2B	9899	0.151986
PPFIA2	8499	0.152439
NEFL	4747	6.548001
TMEM257	9142	0.152855
CXCL14	9547	0.153303
SYNE1	23345	6.508186
NBLA00301	79804	0.153963
LOC441666	441666	6.490143
SOSTDC1	25928	6.488516
KRT33B	3884	6.470345
FUT7	2529	6.454396
SRPX2	27286	6.446732
SEMA3G	56920	0.156687
CCL5	6352	0.156795
GHSR	2693	0.157019
SCN7A	6332	6.367043
UTS2	10911	0.157085
SLCO2B1	11309	6.36024
PNPLA3	80339	6.345309
BRDT	676	6.342818
P2RX1	5023	0.157827
ABCA6	23460	6.322789
GNA15	2769	0.158296
PICK1	9463	6.311152
CFHR5	81494	6.305242
CD44	960	6.300612
IGF1	3479	0.158977
KCNJ4	3761	0.159144
PARVB	29780	6.283343
PLN	5350	0.15949
CCR9	10803	6.262108

TCF4	6925	0.159695
ICAM2	3384	6.212491
TM6SF2	53345	0.162673
PTGES	9536	0.162882
SLC39A9	55334	6.133942
MRC1	4360	0.16316
GFI1	2672	0.164101
SEC14L4	284904	0.164126
GFAP	2670	0.164137
CACNG2	10369	0.164205
MAF	4094	0.164989
ZNF132	7691	6.059267
CDK5R1	8851	0.165179
MLXIP	22877	6.039835
CSAG2///CSAG3	389903///728461	5.990051
HCP5	10866	0.167732
HSD17B2	3294	5.949833
C4BPB	725	5.945282
LRRN3	54674	0.168342
EPHB1	2047	0.168436
KIAA1045	23349	0.168494
GMFG	9535	5.918256
CHM	1121	0.169363
EFNA5	1946	0.169618
CHI3L1	1116	0.16966
FGF2	2247	0.170339
SMPX	23676	5.85365
GPR17	2840	0.171199
MEG3	55384	5.825222
FGF2	2247	0.171904
RHCE	6006	0.172181
KNG1	3827	0.172281
SPATA31C2	645961	0.172453
LALBA	3906	5.784084
ACTL6B	51412	0.173061
C9orf38	29044	5.759905
RAG1	5896	5.756218
CMKLR1	1240	0.173813
POPDC3	64208	0.173852
FKRP	79147	5.747948
MTSS1	9788	5.745005
VCAN	1462	5.739205

6-Sep	23157	0.174491
FABP7	2173	0.174907
ELK1	2002	0.174992
LGALS7///LGALS7B	3963///653499	5.706515
SNAP91	9892	5.696224
LOC100506403///RUNX1	861///100506403	5.69353
LEF1	51176	5.689237
ESR1	2099	5.678731
CIITA	4261	5.671998
GTPBP10	85865	0.176417
HPR	3250	5.653687
FBXO17	115290	5.649747
GPX1	2876	0.177258
SCN3A	6328	0.177369
DDC	1644	5.629318
HLF	3131	0.177756
B3GALT2	8707	0.177996
F13B	2165	0.178002
PRDM10	56980	0.178138
VCAN	1462	0.178211
PRPF31	26121	5.603477
FSHR	2492	5.597511
CDA	978	0.178766
F2RL3	9002	5.582876
RECQL5	9400	0.179167
TAT	6898	5.571299
MGC4294	79160	5.565525
PECAM1	5175	0.179789
TRIM31	11074	0.179794
NPL	80896	0.179887
CES1///LOC100653057	1066///100653057	0.180399
IL7	3574	0.180538
CYP4F2	8529	5.532467
CEACAM21	90273	5.530934
TLL2	7093	0.180842
PRKAA2	5563	0.181181
SMOX	54498	0.181637
FAM5B	57795	0.181702
AVPR2	554	0.182131
U2AF2	11338	0.182384
FAM5C	339479	5.482328

B3GALT1	8708	0.182514
KIAA1661	85375	0.182538
PDCD1LG2	80380	0.182757
KCNB2	9312	0.182843
CEACAM1	634	5.46452
KMT2A	4297	5.464303
NEUROD2	4761	0.183135
COPE	11316	0.18324
POLDIP3	84271	0.183289
BGN	633	5.452267
UGT1A1///UGT1A10///UGT1A3///UGT1A4///UGT1A5///UGT1A6///UGT1A7///UGT1A8///UGT1A9	54575///54576///54577///54578///54579///54600///54657///54658///54659	5.444446
JAK3	3718	0.184047
PPFIA4	8497	5.424242
GPR3	2827	5.391927
ZAP70	7535	5.377475
CADM4	199731	5.371842
CACNA1I	8911	0.186231
CXCL11	6373	5.368142
ACTL7B	10880	0.186855
PTGER1	5731	5.345205
ADCYAP1	116	5.335639
HDAC9	9734	5.333859
KIAA1967	57805	5.332472
AP4E1	23431	5.318138
CLUL1	27098	5.315793
CDKN1C	1028	5.312103
PLCB2	5330	5.302396
RAB40A	142684	5.299368
KCNG1	3755	0.188731
MLLT4	4301	5.294704
DAGLA	747	0.188877
LAPTM5	7805	5.291987
IGLC1	3537	5.288474
CLCN7	1186	5.285575
PCDHGA10///PCDHGA11///PCDHGA12///PCDHGA3///PCDHGA5///PCDHGA6///PCDHGC3	5098///26025///56105///56106///56109///56110///56112	5.281698

PSG5	5673	5.277557
C14orf1	11161	5.253942
BCL11A	53335	0.190902
TRPC3	7222	0.191277
ANGPT2	285	5.227882
PLXNC1	10154	0.191511
BTN2A2	10385	0.191971
DNASE1	1773	5.195785
ATP2A3	489	0.19254
STAB2	55576	0.192576
HOXA7	3204	0.192689
GRIP2	80852	5.174947
DNAJB5	25822	0.193616
CRYBA1	1411	5.153648
MC2R	4158	5.150732
FAM182B///FAM27A///FAM27B///FAM27C	548321///728882///100132948///100133121	0.194155
SPRR1A	6698	0.194298
MYH4	4622	5.137494
MUC4	4585	0.194662
FOLH1	2346	5.133513
MPPE1	65258	0.194899
LAMA4	3910	0.195065
GRM8	2918	5.126296
NOTCH3	4854	5.126225
SLC6A6	6533	5.123775
NR2F6	2063	0.195601
BUB1	699	0.195997
MFAP3L	9848	0.196999
PSG2	5670	5.065988
TRIM15	89870	5.064375
CCL20	6364	0.198221
GPA33	10223	5.039552
C1orf186///LOC100505650	440712///100505650	0.198569
TNMD	64102	0.198714
CBLN1	869	0.198716
LOC101060181///ZNF44	51710///101060181	5.031502
KIR3DL2///LOC727787	3812///727787	0.199451
ADAM3A	1587	5.011363
SCGB1D2	10647	0.199663

SLC26A3	1811	5.007883
LTC4S	4056	0.199985
VGLL1	51442	0.200052
LRRC32	2615	0.200356
N4BP2L1	90634	0.200382
IFNB1	3456	0.200548
LOC100506124///TTC21B	79809///100506124	0.200596
FCER2	2208	4.984489
NCKIPSD	51517	0.200682
TACR1	6869	0.200781
CRTAM	56253	0.20086
IGHV5-78	28387	0.200963
CSN3	1448	4.949852
ZNF154	7710	0.202151
FLT1	2321	0.202276
PDPN	10630	4.940035
FUT3	2525	4.931013
LOC100507472///PCSK6	5046///100507472	0.203107
CPM	1368	4.922866
GPR132	29933	4.922575
PCDHGB6	56100	4.913877
XCL1	6375	0.203546
HIC2	23119	4.90501
MYCN	4613	0.203975
PKD2L2	27039	4.901526
MOGAT2	80168	0.204024
GALR2	8811	4.897788
CEACAM3	1084	0.204564
ID2B	84099	0.205304
SLC24A1	9187	0.205461
CDH6	1004	0.20581
HBE1	3046	4.844616
HCRP1	387535	4.842431
LIPE	3991	0.206557
SLC15A1	6564	0.206567
GPR85	54329	0.206874
SLC7A8	23428	0.207573
TNIK	23043	4.814631
KLK7	5650	0.207762
MASP1	5648	4.812777
WNT5B	81029	0.208045

ERC2-IT1	711	0.208058
KCNAB1	7881	0.2081
CDY1	9085	0.208255
AJAP1	55966	0.208435
C6orf15	29113	0.208688
MTNR1B	4544	0.208781
ESRRG	2104	4.777049
HOXA11	3207	4.77543
CDC42EP3	10602	4.774433
GATA4	2626	4.774418
KCNH1	3756	4.765713
DPT	1805	4.764085
C20orf195	79025	0.210178
DPYSL4	10570	4.747357
DNMT3L	29947	0.210937
MUC7	4589	0.211101
FOXO3///FOXO3B	2309///2310	4.736416
PDE4A	5141	0.211139
TIE1	7075	4.73281
ZNF204P	7754	0.211383
KIR2DL3	3804	0.211516
GULP1	51454	0.211794
BTN3A2	11118	0.211922
CEL	1056	0.211981
F10	2159	4.717063
FEZF2	55079	0.212006
HS3ST1	9957	4.712031
KANSL3	55683	0.212244
CXCR2	3579	0.212573
WNT7B	7477	4.703215
NRXN1	9378	4.698906
CACNA1H	8912	0.212866
NFE2	4778	0.212897
HLA-DRA	3122	4.695531
YME1L1	10730	0.21345
MAP4K2	5871	0.213893
MAGEA10-MAGEA5///MAGEA5	4104///100533997	0.214282
TSHZ2	128553	0.214409
IGH///IGHA2///IGHD///IGHG1	3492///3494///3495///3500	0.21443
OR3A1	4994	4.661118
TRIM46	80128	4.656649

SLC7A8	23428	0.214914
GAGE1	2543	0.215561
CD1D	912	4.623247
GRIN1	2902	0.216345
SLC34A2	10568	0.216387
GNRHR	2798	4.61118
ADD3-AS1	1.01E+08	0.217093
MS4A1	931	4.600435
STAT2	6773	0.217624
TRIM3	10612	4.59444
ENTPD1	953	4.591823
CELF2	10659	4.587314
KLHL35	283212	0.218049
INPP5D	3635	4.582542
COL4A4	1286	0.218448
IPW///LOC100506948///SNORD107///SNORD115-13///SNORD115-26///SNORD115-7///SNORD116-28///SNRPN	3653///6638///91380///100033444///100033450///100033802///100033820///100506948	4.575452
MCHR1	2847	4.573281
PARVB	29780	4.570383
PHYHIP	9796	0.21885
INSL6	11172	4.56706
RFPL1	5988	0.219027
CCL13	6357	0.219075
NR0B2	8431	4.563859
CST1	1469	0.220249
IGFBP3	3486	0.220257
NRG2	9542	4.53621
PDYN	5173	4.534907
CYP4F8	11283	4.531935
GPR12	2835	0.220786
ITGA8	8516	0.220864
VCX2	51480	0.221083
SUGP1	57794	0.221275
ATP8A1	10396	0.22131
KLK14	43847	0.221328
CLCA2	9635	4.514545
CXCR3	2833	4.512124
FBLN1	2192	4.502183
FOXE1	2304	4.492598

OPN1SW	611	4.487854
RPL35A	6165	4.486049
DCHS1	8642	0.223215
GALR1	2587	4.476715
MNDA	4332	0.223473
ICAM1	3383	4.473712
PPP1R3A	5506	0.22368
LTF	4057	0.223887
SAFB2	9667	4.466275
FGFR2	2263	4.455691
ACAA2	10449	0.22453
CHP2	63928	4.451845
KCNJ13	3769	4.448025
CCDC28B	79140	4.447669
MDFIC	29969	4.438573
CLCA1	1179	0.225814
SLC52A1	55065	0.226566
HMGCS2	3158	0.226632
LOC100129973	1E+08	4.406954
G6PC	2538	0.227037
ZNF862	643641	0.22724
PNMA2	10687	0.227415
FCAR	2204	0.227796
JAM2	58494	4.379757
CALD1	800	4.376271
TRIM29	23650	0.228748
SNPH	9751	4.360747
TCF7L2	6934	4.359842
DSCR4	10281	4.354692
TPSAB1	7177	0.229838
CDHR5	53841	0.230038
HFE	3077	0.230202
GFRA2	2675	4.34122
LINC00652	29075	4.333482
GPM6B	2824	4.324861
LZTS1	11178	4.323023
CD5L	922	0.231402
PDE4A	5141	4.318118
CYTIP	9595	0.231725
CLCN4	1183	0.232038
CD84	8832	0.23233
PON3	5446	0.232415
NEK9	91754	0.23262

SNX29	92017	0.232661
IGHA1///IGHA2///IGHG1///IGHG4///IGHM///IGHV4-31	3493///3494///3500///3503///3507///28396	4.297695
CDC42EP4	23580	0.232809
APOL3	80833	0.232852
CDHR5	53841	4.29162
PAX3	5077	0.233321
GART	2618	4.282222
TRIO	7204	4.27639
MYH6	4624	4.272645
MS4A5	64232	0.234066
AHSG	197	4.268815
METTL10	399818	4.26673
LAMB4	22798	0.234445
DOC2B	8447	4.254913
ADRBK1	156	0.235038
CASR	846	0.235447
TAS2R8	50836	0.235545
OR2B6	26212	0.235546
OR12D3///OR5V1	81696///81797	0.235565
ALDOB	229	0.23574
OSM	5008	0.235816
ADAMTS8	11095	0.235901
SNN	8303	0.235994
MAPK8IP3	23162	4.231425
EFCAB6	64800	0.236646
HMOX1	3162	4.208924
SLC6A6	6533	4.208891
ANXA10	11199	4.208301
KLF8	11279	0.237918
BANK1	55024	0.237967
KLRF1	51348	0.238122
HAO1	54363	0.238156
EIF2S2	8894	0.238174
PRMT2	3275	4.197706
HP///HPR	3240///3250	0.238402
LPAR1	1902	0.238816
HRH1	3269	0.238876
FBN1	2200	4.183925
PLIN1	5346	0.239106
CXCL2	2920	0.23943
LCMT2	9836	0.239463

ADAM29	11086	0.239472
AFF3	3899	4.172102
SMARCA1	6594	4.169445
POU2F3	25833	0.239999
OR6A2	8590	0.240191
KMT2A	4297	4.162907
ASIC1	41	0.240353
KRT4	3851	4.158364
TGM2	7052	0.240736
PZP	5858	4.153531
MYH13	8735	0.241222
CYLD	1540	0.241303
C22orf43	51233	4.139056
TGFA	7039	0.241808
SCN2B	6327	4.133081
HGD	3081	4.127912
C1S	716	0.242311
WISP1	8840	4.126851
ERGIC3	51614	0.242881
SLAMF7	57823	4.112216
BAALC	79870	4.11185
HGD	3081	0.243276
TONSL	4796	4.109574
CELA3A	10136	0.243468
COL13A1	1305	0.243818
SOX18	54345	0.244025
ZNF221	7638	0.244298
NID2	22795	0.244729
ALDH1A1	216	0.245012
NCDN	23154	4.073453
NRP2	8828	0.245619
RND1	27289	0.245666
CACNA1E	777	0.245667
CRYAB	1410	4.070503
ALDH3B1	221	0.245739
COL4A1	1282	0.246131
IGLJ3	28831	0.246152
TRMT1	55621	0.246197
IL2RA	3559	4.060918
OSBPL10	114884	0.246424
IL5RA	3568	0.246707
BPY2	9083	4.053234
TRAC///TRAJ17///TRAV20	28663///28738	0.246803

	///28755	
HUWE1	10075	4.048871
PYGO1	26108	0.24699
PPARGC1A	10891	0.247075
GTPBP1	9567	4.039788
GPR15	2838	4.032832
CXorf57	55086	0.248201
TOX3	27324	4.016068
NCLN	56926	0.249145
SLC12A3	6559	4.010562
PIK3R4	30849	0.249425
MMP11	4320	0.249503
ACACB	32	0.249538
IL37	27178	4.006777
RNASE3	6037	4.005261
OGDHL	55753	0.249677
NPTX2	4885	4.003965
CALD1	800	4.001629

Table S6. Content of the 26 bioactive compounds in the SBPpc solution

NO.	Compound	Content (mg)
S-1	Muscone	1.05
S-2	Bufalin	4.95
S-3	Resibufogenin	3.33
S-4	Cinobufogenin/cinobufagin	0.64
S-5	Gamabufagin	0.63
S-6	Arenobufagin	0.62
S-7	Telocinobufagin	0.09
S-8	Bufotalin	0.23
S-9	Cinbufotalin	0.15
S-10	Cholic acid	5.1
S-11	Deoxycholic acid	0.88
S-12	Chenodeoxycholic acid	0.57
S-13	Hyodeoxycholic acid	0.44
S-14	Ursodeoxycholic acid	1.48
S-15	Cinnamic aldehyde	1.43
S-16	Cinnamic acid	0.36
S-17	Ginsenoside Rb1	7.65
S-18	Ginsenoside Rb2	4.64
S-19	Ginsenoside Rb3	2.43

S-20	Ginsenoside Rc	7.26
S-21	Ginsenoside Rd	4.73
S-22	Ginsenoside Re	7.27
S-23	Ginsenoside Rg1	8.75
S-24	Borneol	82.37
S-25	(+)-Borneol	31.63
S-26	Benzyl benzonate	8.37

Table S7. The 63 experimentally validated CVD pathways regulated by both SBPac and SBPpc

Pathway ID	Pathway name	Pathway url
636	PID_LYMPHANGIOGENESIS_PATHWAY	http://www.broadinstitute.org/gsea/msigdb/cards/PID_LYMPHANGIOGENESIS_PATHWAY
630	PID_S1P_S1P2_PATHWAY	http://www.broadinstitute.org/gsea/msigdb/cards/PID_S1P_S1P2_PATHWAY
613	PID_SYNDECAN_2_PATHWAY	http://www.broadinstitute.org/gsea/msigdb/cards/PID_SYNDECAN_2_PATHWAY
601	PID_VEGFR1_PATHWAY	http://www.broadinstitute.org/gsea/msigdb/cards/PID_VEGFR1_PATHWAY
595	PID_AR_NONGENOMIC_PATHWAY	http://www.broadinstitute.org/gsea/msigdb/cards/PID_AR_NONGENOMIC_PATHWAY
574	PID_ECADHERIN_KERATINOCYTE_PATHWAY	http://www.broadinstitute.org/gsea/msigdb/cards/PID_ECADHERIN_KERATINOCYTE_PATHWAY
567	PID_ERBB2ERBB3PATHWAY	http://www.broadinstitute.org/gsea/msigdb/cards/PID_ERBB2ERBB3PATHWAY
548	PID_IL2_PI3KPATHWAY	http://www.broadinstitute.org/gsea/msigdb/cards/PID_IL2_PI3KPATHWAY
536	PID_ERBB1_RECEPTOR_PROXIMAL_PATHWAY	http://www.broadinstitute.org/gsea/msigdb/cards/PID_ERBB1_RECEPTOR_PROXIMAL_PATHWAY
533	PID_IL2_1PATHWAY	http://www.broadinstitute.org/gsea/msigdb/cards/PID_IL2_1PATHWAY
521	PID_S1P_S1P1_PATHWAY	http://www.broadinstitute.org/gsea/msigdb/cards/PID_S1P_S1P1_PATHWAY
515	PID_ANGIOPOIETINRECEPTOR_PATHWAY	http://www.broadinstitute.org/gsea/msigdb/cards/PID_ANGIOPOIETINRECEPTOR_PATHWAY
493	PID_AVB3_OPN_PATHWAY	http://www.broadinstitute.org/gsea/msigdb/cards/PID_AVB3_OPN_PATHWAY
409	SA_B_CELL_RECEPTOR_COMPLEXES	http://www.broadinstitute.org/gsea/msigdb/cards/SA_B_CELL_RECEPTOR_COMPLEXES
4030	TCGA_GLIOBLASTOMA_MUTATED	http://www.broadinstitute.org/gsea/msigdb/cards/TCGA_GLIOBLASTOMA_MUTATED
4028	DING_LUNG_CANCER_MUTATED_RECURRENTLY	http://www.broadinstitute.org/gsea/msigdb/cards/DING_LUNG_CANCER_MUTATED_RECURRENTLY
398	BIOCARTA_ARF_PATHWAY	http://www.broadinstitute.org/gsea/msigdb/cards/BIOCARTA_ARF_PATHWAY
397	BIOCARTA_TRKA_PATHWAY	http://www.broadinstitute.org/gsea/msigdb/cards/BIOCARTA_TRKA_PATHWAY
396	BIOCARTA_TFF_PATHWAY	http://www.broadinstitute.org/gsea/msigdb/cards/BIOCARTA_TFF_PATHWAY
394	BIOCARTA_CREB_PATHWAY	http://www.broadinstitute.org/gsea/msigdb/cards/BIOCARTA_CREB_PATHWAY
393	BIOCARTA_TPO_PATHWAY	http://www.broadinstitute.org/gsea/msigdb/cards/BIOCARTA_TPO_PATHWAY
381	BIOCARTA_TGFB_PATHWAY	http://www.broadinstitute.org/gsea/msigdb/cards/BIOCARTA_TGFB_PATHWAY
366	BIOCARTA_MET_PATHWAY	http://www.broadinstitute.org/gsea/msigdb/cards/BIOCARTA_MET_PATHWAY
353	BIOCARTA_ERK5_PATHWAY	http://www.broadinstitute.org/gsea/msigdb/cards/BIOCARTA_ERK5_PATHWAY

3521	BIERIE_INFLAMMATORY_RESPONSE_TGFB1	http://www.broadinstitute.org/gsea/msigdb/cards/BIERIE_INFLAMMATORY_RESPONSE_TGFB1
352	BIOCARTA_HER2_PATHWAY	http://www.broadinstitute.org/gsea/msigdb/cards/BIOCARTA_HER2_PATHWAY
351	BIOCARTA_CARDIACEGF_PATHWAY	http://www.broadinstitute.org/gsea/msigdb/cards/BIOCARTA_CARDIACEGF_PATHWAY
331	BIOCARTA_PTEN_PATHWAY	http://www.broadinstitute.org/gsea/msigdb/cards/BIOCARTA_PTEN_PATHWAY
321	BIOCARTA_CCR5_PATHWAY	http://www.broadinstitute.org/gsea/msigdb/cards/BIOCARTA_CCR5_PATHWAY
3118	WU_HBX_TARGETS_3_DN	http://www.broadinstitute.org/gsea/msigdb/cards/WU_HBX_TARGETS_3_DN
311	BIOCARTA_NTHI_PATHWAY	http://www.broadinstitute.org/gsea/msigdb/cards/BIOCARTA_NTHI_PATHWAY
308	BIOCARTA_NGF_PATHWAY	http://www.broadinstitute.org/gsea/msigdb/cards/BIOCARTA_NGF_PATHWAY
299	BIOCARTA_EGFR_SMRTE_PATHWAY	http://www.broadinstitute.org/gsea/msigdb/cards/BIOCARTA_EGFR_SMRTE_PATHWAY
298	BIOCARTA_PYK2_PATHWAY	http://www.broadinstitute.org/gsea/msigdb/cards/BIOCARTA_PYK2_PATHWAY
292	BIOCARTA_INSULIN_PATHWAY	http://www.broadinstitute.org/gsea/msigdb/cards/BIOCARTA_INSULIN_PATHWAY
291	BIOCARTA_GLEEVEC_PATHWAY	http://www.broadinstitute.org/gsea/msigdb/cards/BIOCARTA_GLEEVEC_PATHWAY
290	BIOCARTA_RACCYCD_PATHWAY	http://www.broadinstitute.org/gsea/msigdb/cards/BIOCARTA_RACCYCD_PATHWAY
282	BIOCARTA_IL6_PATHWAY	http://www.broadinstitute.org/gsea/msigdb/cards/BIOCARTA_IL6_PATHWAY
280	BIOCARTA_IL4_PATHWAY	http://www.broadinstitute.org/gsea/msigdb/cards/BIOCARTA_IL4_PATHWAY
276	BIOCARTA_IGF1_PATHWAY	http://www.broadinstitute.org/gsea/msigdb/cards/BIOCARTA_IGF1_PATHWAY
275	BIOCARTA_HIF_PATHWAY	http://www.broadinstitute.org/gsea/msigdb/cards/BIOCARTA_HIF_PATHWAY
273	BIOCARTA_HCMV_PATHWAY	http://www.broadinstitute.org/gsea/msigdb/cards/BIOCARTA_HCMV_PATHWAY
250	BIOCARTA_EPO_PATHWAY	http://www.broadinstitute.org/gsea/msigdb/cards/BIOCARTA_EPO_PATHWAY
246	BIOCARTA_EGF_PATHWAY	http://www.broadinstitute.org/gsea/msigdb/cards/BIOCARTA_EGF_PATHWAY
2288	GALIE_TUMOR_ANGIOGENESIS	http://www.broadinstitute.org/gsea/msigdb/cards/GALIE_TUMOR_ANGIOGENESIS
214	BIOCARTA_CDMAC_PATHWAY	http://www.broadinstitute.org/gsea/msigdb/cards/BIOCARTA_CDMAC_PATHWAY
2083	AGARWAL_AKT_PATHWAY_TARGETS	http://www.broadinstitute.org/gsea/msigdb/cards/AGARWAL_AKT_PATHWAY_TARGETS
207	BIOCARTA_BCELLSURVIVAL_PATHWAY	http://www.broadinstitute.org/gsea/msigdb/cards/BIOCARTA_BCELLSURVIVAL_PATHWAY
204	BIOCARTA_SPPA_PATHWAY	http://www.broadinstitute.org/gsea/msigdb/cards/BIOCARTA_SPPA_PATHWAY
199	BIOCARTA_AT1R_PATHWAY	http://www.broadinstitute.org/gsea/msigdb/cards/BIOCARTA_AT1R_PATHWAY
1956	DEBOSSCHER_NFKB_TARGETS_REPRESSED_BY_GLUCOCORTICOIDS	http://www.broadinstitute.org/gsea/msigdb/cards/DEBOSSCHER_NFKB_TARGETS_REPRESSED_BY_GLUCOCORTICOIDS
1954	SCHEIDEREIT_IKK_TARGETS	http://www.broadinstitute.org/gsea/msigdb/cards/SCHEIDEREIT_IKK_TARGETS
1942	BAKER_HEMATOPOIESIS_STAT3_TARGETS	http://www.broadinstitute.org/gsea/msigdb/cards/BAKER_HEMATOPOIESIS_STAT3_TARGETS
1941	BAKER_HEMATOPOESIS_STAT1_TARGETS	http://www.broadinstitute.org/gsea/msigdb/cards/BAKER_HEMATOPOESIS_STAT1_TARGETS
1932	TURJANSKI_MAPK14_TARGETS	http://www.broadinstitute.org/gsea/msigdb/cards/TURJANSKI_MAPK14_TARGETS
1930	TURJANSKI_MAPK7_TARGETS	http://www.broadinstitute.org/gsea/msigdb/cards/TURJANSKI_MAPK7_TARGETS
1929	TURJANSKI_MAPK8_AND_MAPK9_TARGETS	http://www.broadinstitute.org/gsea/msigdb/cards/TURJANSKI_MAPK8_AND_MAPK9_TARGETS
1928	TURJANSKI_MAPK1_AND_MAPK2_TARGETS	http://www.broadinstitute.org/gsea/msigdb/cards/TURJANSKI_MAPK1_AND_MAPK2_TARGETS
1891	MARKS_HDAC_TARGETS_DN	http://www.broadinstitute.org/gsea/msigdb/cards/MARKS_HDAC_TARGETS_DN

1851	BUSA_SAM68_TARGETS_DN	http://www.broadinstitute.org/gsea/msigdb/cards/BUSA_SAM68_TARGETS_DN
172	KEGG_BLADDER_CANCER	http://www.broadinstitute.org/gsea/msigdb/cards/KEGG_BLADDER_CANCER
165	KEGG_PANCREATIC_CANCER	http://www.broadinstitute.org/gsea/msigdb/cards/KEGG_PANCREATIC_CANCER
1088	REACTOME_ACTIVATION_OF_THE_AP1_FAMILY_OF_TRANSCRIPTION_FACTORS	http://www.broadinstitute.org/gsea/msigdb/cards/REACTOME_ACTIVATION_OF_THE_AP1_FAMILY_OF_TRANSCRIPTION_FACTORS
